# Subclinical Coronary Atherosclerosis and Retinal Optical Coherence Tomography Angiography

**DOI:** 10.1001/jamacardio.2025.3036

**Published:** 2025-09-17

**Authors:** Jee Myung Yang, Dong Hyun Yang, Seung-Whan Lee, Jiehoon Kwak, Yunhan Lee, Yoon Jeon Kim, Joo Yong Lee, Kyung Rim Sung, Young Hee Yoon

**Affiliations:** 1 Department of Ophthalmology, Asan Medical Center, University of Ulsan College of Medicine, Seoul, South Korea; 2 Department of Radiology, Asan Medical Center, University of Ulsan College of Medicine, Seoul, South Korea; 3 Department of Cardiology, Asan Medical Center, University of Ulsan College of Medicine, Seoul, South Korea; 4 Graduate School of Medical Science and Engineering, Korea Advanced Institute of Science and Technology (KAIST), Daejeon, South Korea

## Abstract

**Importance:**

Systemic disease burden is well reflected in the retinal microvasculature, but it is not established whether optical coherence tomography angiography (OCTA) parameters can reliably reflect coronary atherosclerosis.

**Objective:**

To assess the association of reduced retinal vascular density and subclinical coronary atherosclerosis in asymptomatic individuals.

**Design, Setting, and Participants:**

This cross-sectional cohort study included asymptomatic individuals with elevated cardiovascular risk who self-referred for a health screening program involving coronary computed tomography angiography (CTA) at Asan Medical Center in Seoul, South Korea, and subsequently underwent OCTA as part of ophthalmic evaluations. This study was conducted from October 2015 to December 2020, and data analysis was conducted from January 2021 to May 2025.

**Main Outcomes and Measures:**

The primary outcome was the association between OCTA parameters and coronary CTA parameters.

**Results:**

A total of 1286 eyes from 1286 participants were analyzed (mean [SD] age, 64.2 [9.9] years; 482 female participants [37.5%]). Coronary artery calcium score (CACS), presence of plaque and its subtypes, obstructive coronary artery disease (CAD), severe CAD, segment stenosis score (SSS), and segment involvement score (SIS) were significantly increased across the quartiles of superficial and deep parafoveal vascular density (PFVD). When modeled as continuous variables, superficial capillary plexus (SCP) and deep capillary plexus (DCP) PFVD were the most significant metrics and correlated with CACS, number of coronary vessels involved, SSS, and SIS. The lowest quartile of SCP PFVD was associated with significantly higher odds of obstructive CAD (adjusted odds ratio [aOR], 2.91; 95% CI, 1.83-4.73), severe CAD (aOR, 3.30; 95% CI, 1.55-7.91), and elevated SSS and SIS scores compared to the highest quartile. Similar but attenuated associations were observed for DCP PFVD. Continuous variable analysis for ORs for unit increase supported a linear inverse association between PFVD and CAD burden. Incorporating PFVD into models with cardiovascular risk factors improved area under the curve (AUC) for identifying severe CAD (AUC, 0.79; 95% CI, 0.75-0.82), obstructive CAD (AUC, 0.78; 95% CI, 0.76-0.81), and SSS of 10 or higher (AUC, 0.77; 95% CI, 0.74-0.80), with SCP yielding superior performance over DCP.

**Conclusions and Relevance:**

In this cross-sectional cohort study, reduced retinal PFVD was independently associated with subclinical coronary atherosclerosis in a population with elevated vascular risk. In this context, decreased PFVD may reflect greater subclinical coronary atherosclerotic burden and help identify individuals who could benefit from further coronary evaluation, beyond traditional risk factors.

## Key Points

**Question:**

Can evaluation of retinal microvasculature by optical coherence tomography angiography (OCTA) serve as a diagnostic tool for detecting subclinical coronary atherosclerosis?

**Findings:**

In this clinic-based cross-sectional cohort study that included 1286 patients who underwent both coronary computed tomography angiography (CTA) and OCTA, coronary artery calcium score, presence of plaque, obstructive coronary artery disease (CAD), severe CAD, segment stenosis score, and segment involvement score were significantly associated with parafoveal vascular density (PFVD).

**Meaning:**

Reduced retinal PFVD was associated with subclinical coronary atherosclerosis and, alongside cardiovascular risk factors, may help identify patients needing comprehensive coronary screening.

## Teaser

This cross-sectional cohort study demonstrates the association of reduced retinal vascular density and subclinical coronary atherosclerosis in asymptomatic individuals.

## Introduction

Coronary computed tomography angiography (CTA) is a powerful noninvasive tool for detecting subclinical atherosclerosis.^1,2,3^ While coronary CTA benefits early detection of subclinical atherosclerosis in asymptomatic individuals, identifying at-risk candidates remains challenging. Accumulating evidence indicates that changes in superficial capillary plexus (SCP) and deep capillary plexus (DCP) of the retina are strongly associated with impaired systemic microcirculation.^4,5,6^ Furthermore, the retinal microvasculature exhibits physiological and anatomical characteristics comparable to those of the coronary system.^7^ Thus, evaluating the retinal microvasculature may provide a valuable biomarker to improve coronary artery disease (CAD) risk stratification and facilitate the early detection of subclinical atherosclerosis.

Optical coherence tomography angiography (OCTA) is a sensitive, noninvasive, state-of-the-art method for directly visualizing retinal microvasculature.^8^ OCTA enables detailed analysis of the retinal vasculature by robustly quantifying foveal and parafoveal vascular density (VD) in the superficial and deep retinal layers, as well as the foveal avascular zone (FAZ), through internal software programs.^8^ Previous studies have reported decreased retinal VD in patients with CAD.^9,10,11^ While insightful, these studies warrant larger, well-adjusted cohorts to better clarify the CAD–retinal microvasculature association. Additionally, to our knowledge no studies to date have evaluated the association between OCTA and coronary CTA parameters. Given that systemic disease burden is well reflected in the retinal microvasculature, which can be assessed in detail by OCTA,^7,12,13^ we hypothesize that OCTA parameters may serve as effective supporting tool for coronary atherosclerosis diagnosis. Using noninvasive OCTA, we aimed to analyze the correlation between retinal microvascular changes and coronary artery status evaluated by coronary CTA and to identify retinal indicators that may aid in the diagnosis of CAD in a large cohort of asymptomatic Korean individuals.

## Methods

### Study Population

In this cross-sectional study, from October 2015 to December 2020, 2379 asymptomatic participants underwent both coronary CTA as part of a self-referred health screening program at the Asan Medical Center in Seoul, South Korea, and review of OCTA results for detailed retinal disorder screening and glaucoma (eTable 1 in Supplement 1) as part of a routine tertiary center protocol (eFigure 1 in Supplement 1). This study was based on a newly established registry, and asymptomatic participants were included consecutively during the study period, with exclusions applied based on predefined quality and data completeness criteria. All participants were informed of the potential risks of undergoing coronary CTA and provided written informed consent. Participants were excluded if they met any of the following criteria: (1) age younger than 18 years; (2) history of angina or myocardial infarction; (3) abnormal coronary structure due to open heart surgery or percutaneous coronary intervention; or (4) insufficient medical records.^14^ OCTA images closest to the coronary CTA examination date (within 12 months) were selected for analysis.

For OCTA evaluation, the eye with the worst best-corrected visual acuity (BCVA) was selected. If both eyes had the same BCVA, the right eye was chosen. Fellow eyes were studied when the eyes had a history of significant macular disorder. Eyes with a history of intravitreal injection, vitrectomy, or ocular disorders, such as macular edema, vitreous hemorrhage, or trauma that induced segmentation error of the OCTA and had a signal strength index (SSI) of less than 50, were excluded. Additionally, participants with high myopia (spherical equivalent >6.0 diopters or axial length >26 mm) and with a history of significant bilateral macular diseases, intraocular infection, uveitis, trauma, or glaucoma were excluded. Comparison of BCVA between included and excluded participants showed no significant difference, indicating minimal selection bias related to visual function. Finally, a total of 1286 eyes from 1286 participants were included. The study was approved by the institutional review board and ethics committee of Asan Medical Center, Seoul, Korea (2021-0018).

### Clinical and Laboratory Measurements

Clinical and laboratory data and information on smoking history, comorbidities, medications, and physical findings closest to the coronary CTA date were collected through medical records review. Biochemical tests were obtained using standardized methods at a single accredited laboratory (Asan Medical Center, Seoul, South Korea).

### Coronary CTA Image Acquisition and Analysis

All participants underwent multidetector coronary CTA using a second- or third-generation dual-source scanner (SOMATOM Definition [Siemens]) following standard scanning protocol, as previously described.^1,15^ All coronary CTA images were interpreted by 3 board-certified radiologists based on the established interpretation guidelines, and a cardiovascular radiologist (D.H.Y.) summarized the results based on their interpretations.^16^ Obstructive CAD with significant stenosis was defined as stenosis of 50% or higher. The total atherosclerotic plaque burden was assessed using a coronary artery plaque score: segment involvement score (SIS) and segment stenosis score (SSS). Severe CAD was defined as meeting 1 of the following criteria: (1) ≥2-vessel coronary disease involving the proximal left anterior descending coronary artery (LAD); (2) 3-vessel disease; or (3) left main coronary artery (LM) disease.^17^

### OCTA Analysis

OCTA images were acquired using the Optovue RTVue XR Avanti (Optovue) with AngioVue software (versions 2016.2.0.35 to 2018.1.0.37), as previously described.^18^ Briefly, macular cube scans (3 × 3 mm) containing 304 clusters of 2 repeated B-scans, each comprising 304 A-scans, were acquired. The SCP was segmented from 3 µm below the internal limiting membrane to 15 µm below the inner plexiform layer, and the DCP from 15 µm to 70 µm below the inner nuclear layer to outer plexiform layer, using the device’s default settings.^19^ All OCTA images were reviewed B-scan by B-scan by trained graders (J.K. and Y.L.) evaluating the segmentation lines of the SCP and DCP to detect segmentation errors by using AngioVue software (AngioRetina mode). In cases of incorrect segmentation, manual adjustment of the boundary was performed.^20^ The following retinal microvascular parameters were measured: FAZ area (in millimeters squared), SCP, DCP vessel density (VD, percentage) of the foveal area (1-mm diameter, central subfield of Early Treatment Diabetic Retinopathy Study [ETDRS] grid sector), VD (percentage) of the parafovea (averaged from 4 subfield of the inner ring area with a 3-mm diameter, excluding central subfield), and central retinal thickness (inner limiting membrane to retinal pigment epithelium layers).^21^ Patients’ dilation status did not introduce meaningful bias, but images with low quality (SSI <50), poor fixation-induced artifacts, and failure of layer segmentation were excluded from the analysis. Images with uncorrectable artifacts or subclinical pathology affecting segmentation were excluded from analysis.

### Statistical Analysis

We performed a complete-case analysis. Participants with missing data on cardiovascular risk factors or key clinical covariates were excluded (eFigure 1 in Supplement 1). Baseline characteristics of excluded and included participants were compared, and no significant differences were observed (eTable 2 in Supplement 1), suggesting that data were missing at random. The distribution of continuous variables was assessed using the Shapiro-Wilk test for normality. Categorical variables are presented as proportions (percentages) and compared using χ^2^ tests. Variables that followed a normal distribution were presented as mean and standard deviation, while those with non-normal distributions were reported as median with interquartile range. Group comparisons for continuous variables were performed using *t* tests (or analysis of variance for >2 groups) or Mann-Whitney *U* test (or Kruskal-Wallis test for >2 group), as appropriate. Pearson correlation analysis was conducted to assess the correlation between continuous variables. Linearity was assessed using scatterplots with locally estimated scatterplot smoothing (LOESS) curves and comparison of Pearson and Spearman correlation coefficients. The area under the curve (AUC) of the receiver operating characteristics (ROC) curve was analyzed to evaluate the model’s discriminative ability in discriminating cases of subclinical atherosclerosis cases from noncases. To assess significant improvement in AUC values, the DeLong test for 2 correlated ROC curves was assessed. The optimal cutoff value for SCP and DCP parafoveal VD (PFVD), as well as for other ROC-based models, were identified using the Youden index. The PFVD was divided into quartiles (lowest VD [quartile 1] to highest VD [quartile 4]) for analysis. Dichotomization of continuous outcome variables (coronary artery calcium score [CACS], SSS, and SIS) were based on clinically accepted thresholds to facilitate interpretability and clinical relevance.^22,23^ Logistic regression models were used to calculate odds ratios (ORs) with 95% confidence intervals adjusted for confounders as indicated.^24,25^ Existing risk factors were adjusted by using the atherosclerotic cardiovascular disease (ASCVD) pooled cohort equation in the primary models.^25^ A sensitivity analysis incorporating the PREVENT risk score was conducted among participants aged 30 to 79 years.^26^ A sensitivity analysis in a subset of patients with diabetes was also conducted. To minimize multicollinearity, variables with variance inflation factor (≥5) were not included simultaneously as covariates, but analyzed separately when serving as outcome variables. Multiple comparisons were adjusted using the Benjamini-Hochberg procedure to control the false discovery rate (FDR). All analyses were performed using SAS version 9.4 (SAS Institute) and R version 4.3.3 (R Foundation). A *P* value <.05 was considered statistically significant.

More details of the methods, including statistical analysis, are described in the eMethods in Supplement 1.

## Results

### Population Characteristics

Clinical characteristics of the study participants are described in Table 1. Mean (SD) participant age was 64.2 (9.9) years, with 482 female participants (37.5%). Among the participants, 136 (10.6) had a CACS greater than 400, 804 (62.5%) had any plaque, 300 (23.3%) had obstructive CAD, and 120 (9.3%) had severe CAD.

**Table 1.** Baseline Clinical Characteristics of the Participants

Baseline characteristic	Overall (N = 1286), No. (%)
Age, mean (SD), y	64.2 (9.9)
Age category, y	
>55	205 (15.9)
55-70	696 (54.1)
>70	385 (29.9)
Sex	
Female	482 (37.5)
Male	804 (62.5)
BMI^a^	
Median (IQR)	24.60 (22.64-26.56)
BMI category	
<25	709 (55.1)
25-30	503 (39.1)
≥30	74 (5.8)
Smoking	
Current	135 (10.5)
Never	802 (62.4)
Former	349 (27.1)
Systolic blood pressure, mean (SD), mm Hg	130.19 (10.04)
ASCVD risk, mean (SD), %	12.22 (14.68)
ASCVD risk category	
Low (<5%)	575 (44.7)
Borderline (5%-7.5%)	73 (5.7)
Intermediate (>7.5%-20%)	331 (25.7)
High (≥20%)	307 (23.9)
Comorbidities	
Diabetes	552 (42.9)
Hypertension	675 (52.5)
Hyperlipidemia	930 (72.5)
Stroke	80 (6.2)
Kidney disease	94 (7.3)
Medications	
Aspirin	260 (20.2)
Clopidogrel	337 (26.2)
Statin	803 (62.4)
Insulin	108 (8.4)
Metformin	376 (29.2)
Laboratory parameters	
HbA_1c_, mean (SD), %	6.25 (1.18)
HbA_1c_ category	
<7%	992 (77.1)
≥7%	294 (22.9)
Fasting glucose, mean (SD), mg/dL	124.21 (43.04)
Triglycerides, mean (SD), mg/dL	129.33 (75.73)
HDL, mean (SD), mg/dL	50.27 (14.02)
LDL, mean (SD), mg/dL	102.85 (32.49)
CRP, mean (SD), mg/dL	0.80 (2.35)
eGFR, median (IQR), mL/min/1.73 m^2^	85.00 (71.25-95.00)
eGFR category, mL/min/1.73 m^2^	
≤59	147 (11.4)
60-89	621 (48.3)
≥90	518 (40.3)
Ophthalmic and OCTA parameters, mean (SD)	
BCVA, logMAR	0.37 (0.44)
FAZ size, mm^2^	0.35 (0.48)
SCP foveal VD, %	18.95 (10.16)
SCP parafoveal VD, %	45.00 (7.32)
DCP fovea VD, %	32.28 (10.04)
DCP parafoveal VD, %	49.08 (7.16)
CFT, µm	264.35 (63.44)
Signal strength	63.24 (6.86)
Interval between OCTA and coronary CTA, median (IQR), mo	4.4 (2.0-7.7)
Coronary CTA parameters	
Coronary artery calcium score, median (IQR)	0.00 (0.00-98.70)
Coronary artery calcium score category	
0	666 (51.8)
1-10	105 (8.2)
11-100	196 (15.2)
101-400	183 (14.2)
>400	136 (10.6)
Any plaque	804 (62.5)
Plaque characteristics	
Calcified plaque	685 (53.3)
Noncalcified plaque	234 (18.2)
Mixed plaque	320 (24.9)
Obstructive CAD	300 (23.3)
No. of obstructive CAD lesions	
1 Vessel	148 (11.5)
2 Vessels	88 (6.8)
3 Vessels	63 (4.9)
LM obstruction	23 (1.8)
Obstructive CAD in the LM or pLAD	
None	1141 (88.7)
Single	126 (9.8)
Both	19 (1.5)
Severe CAD	120 (9.3)
Segment stenosis score, mean (SD)	5.10 (7.18)
Segment stenosis score ≥10	261 (20.3)
Segment involvement score, mean (SD)	2.53 (2.81)
Segment involvement score ≥5	220 (17.1)

Abbreviations: ASCVD, atherosclerotic cardiovascular disease; BCVA, best-corrected visual acuity; BMI, body mass index; CAD, coronary artery disease; CFT, central foveal thickness; CRP, C-reactive protein; CTA, computed tomography angiography; DCP, deep capillary plexus; eGFR, estimated glomerular filtration rate; FAZ, foveal avascular zone; HbA_1c_, hemoglobin A_1c_ (glycated hemoglobin); HDL, high-density lipoprotein; LDL, low-density lipoprotein; LM, left main; logMAR, logarithm of the minimum angle of resolution; OCTA, optical coherence tomography angiography; pLAD, proximal left anterior descending; SCP, superficial capillary plexus; VD, vascular density.

SI conversion factors: To convet HbA_1c_ from percentage of total hemoglobin to proportion of total hemoglobin, multiply by 0.01; fasting glucose, from mg/dL to mmol/L, multiply by 0.0555; triglycerides, from mg/dL to mmol/L, multiply by 0.0113; HDL and LDL, from mg/dL to mmol/L, multiply by 0.0259; CRP, from mg/dL to mg/L, multiply by 10.

^a^ Calculated as weight in kilograms divided by height in meters squared.

### Association Between Retinal PFVD and Subclinical Atherosclerosis

A correlation analysis between OCTA parameters and continuous coronary CTA variables was performed (eFigures 2-4 in Supplement 1). SCP and DCP PFVD showed the strongest significant correlations with CACS, the number of coronary vessels involved, SSS, and SIS (eTable 3 in Supplement 1). Additionally, SCP and DCP PFVD showed a significant negative correlation with SSS and SIS. Consequently, SCP and DCP PFVD were selected for a detailed analysis of their association with subclinical CAD.

**Figure 1.**
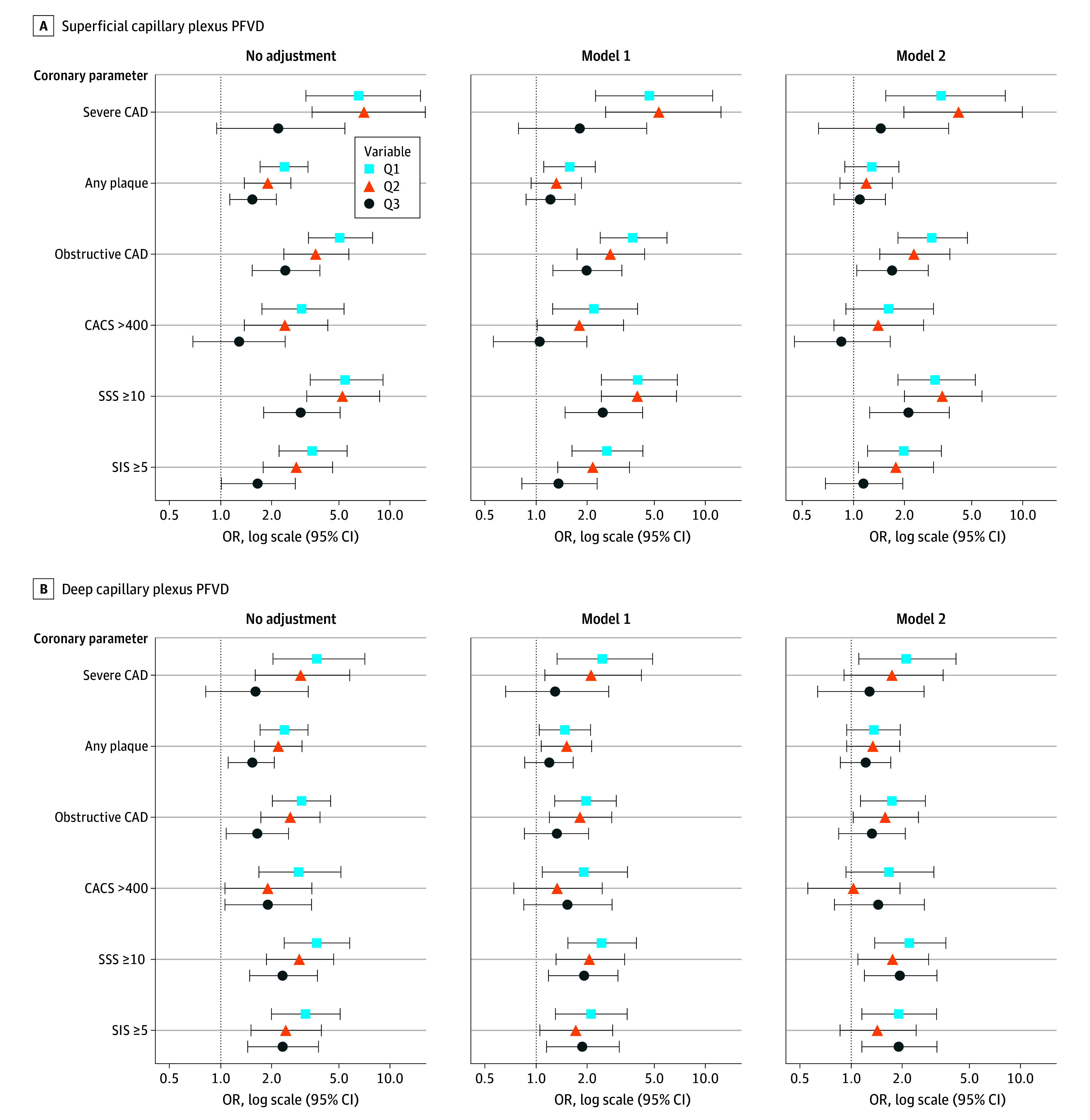
Association Between Retinal Parafoveal Vascular Density and Subclinical Coronary Atherosclerosis Logistic regression analyses using quartile (Q) of superficial capillary plexus parafoveal vascular density (PFVD) (A) and deep capillary plexus PFVD (B). Reference is Q4. CACS indicates coronary artery calcium score; CAD, coronary artery disease; OR, odds ratio; SIS, segment involvement score; SSS, segment stenosis score.

Table 2 and eTable 4 in Supplement 1 show the baseline characteristics of participants by quartiles of SCP and DCP PFVD. Lower PFVD was significantly associated with higher CACS, a greater number of plaques, obstructive CAD, an increased likelihood of obstructive CAD in the LM or proximal LAD, a higher number of severe CAD, and higher SSS and SIS scores (all FDR <0.05). The odds of subclinical atherosclerosis are shown in Figure 1 and in eFigure 5 and eTables 5 to 7 in Supplement 1. Using quartile 4 (highest VD) as a reference, quartile 1 (lowest VD) for SCP PFVD showed significantly higher odds of obstructive CAD (OR, 2.91; 95% CI, 1.83-4.73), severe CAD (OR, 3.30; 95% CI, 1.55-7.91), SSS of 10 or higher (OR, 3.04; 95% CI, 1.82-5.26), and SIS of 5 or higher (OR, 1.97; 95% CI, 1.20-3.30) after multivariable adjustment. Similarly, in quartile 1 for DCP PFVD, the odds of obstructive CAD (OR, 1.75; 95% CI, 1.13-2.72) and severe CAD (OR, 2.10; 95% CI, 1.11-4.17) were higher with multivariable adjustment. Continuous variable analysis and distribution plot confirmed a linear association between decreasing PFVD and increasing CAD burden (eFigures 5 and 6 and eTable 6 in Supplement 1). A sensitivity analysis incorporating the PREVENT risk score yielded consistent results compared to full cohort (eFigure 7 and eTable 8 in Supplement 1).

**Table 2.** Coronary Computed Tomography Angiography (CTA) Parameters by Retinal Parafoveal Vascular Density (PFVD) Quartile (Q)

	SCP PFVD						DCP PFVD					
	No. (%)				*P *value	FDR^a^	No. (%)				*P* value	FDR^a^
	Q4 (Highest)	Q3	Q2	Q1 (Lowest)			Q4 (Highest)	Q3	Q2	Q1 (Lowest)		
No.	322	322	321	321	NA	NA	322	322	321	321	NA	NA
SCP PFVD, mean (SD), %	53.3 (2.0)	48.1 (1.3)	43.4 (1.56)	35.1 (5.2)	NA	NA	49.8 (5.7)	46.5 (5.7)	44.1 (5.9)	39.6 (7.8)	NA	NA
DCP PFVD, mean (SD), %	53.9 (5.0)	50.2 (4.5)	48.0 (6.1)	44.2 (8.7)	NA	NA	56.8 (2.3)	51.6 (1.2)	47.8 (1.1)	40.0 (6.9)	NA	NA
Age, mean (SD), y	59.8 (8.89)	62.7 (9.8)	66.8 (9.6)	67.7 (9.2)	<.001	<0.001	60.3 (9.56)	63.7 (9.2)	65.7 (10.4)	67.2 (9.2)	<.001	<0.001
Age category, y												
<55	93 (28.9)	59 (18.3)	29 (9.0)	24 (7.5)	<.001	<0.001	87 (27.0)	53 (16.5)	44 (13.7)	21 (6.5)	<.001	<0.001
55-70	190 (59.0)	181 (56.2)	172 (53.6)	153 (47.7)			186 (57.8)	186 (57.8)	158 (49.2)	166 (51.7)		
>70	39 (12.1)	82 (25.5)	120 (37.4)	144 (44.9)			49 (15.2)	83 (25.8)	119 (37.1)	134 (41.7)		
Sex												
Female	136 (42.2)	105 (32.6)	119 (37.1)	122 (38.0)	.09	0.17	154 (47.8)	120 (37.3)	102 (31.8)	106 (33.0)	<.001	<0.001
Male	186 (57.8)	217 (67.4)	202 (62.9)	199 (62.0)			168 (52.2)	202 (62.7)	219 (68.2)	215 (67.0)		
Smoking												
Current	36 (11.2)	42 (13.0)	26 (8.1)	31 (9.7)	.33	0.36	41 (12.7)	30 (9.3)	42 (13.1)	22 (6.9)	.06	0.10
Never	203 (63.0)	187 (58.1)	213 (66.4)	199 (62.0)			208 (64.6)	199 (61.8)	190 (59.2)	205 (63.9)		
Former	83 (25.8)	93 (28.9)	82 (25.5)	91 (28.3)			73 (22.7)	93 (28.9)	89 (27.7)	94 (29.3)		
Diabetes	94 (29.2)	139 (43.2)	150 (46.7)	169 (52.6)	<.001	<0.001	102 (31.7)	128 (39.8)	163 (50.8)	159 (49.5)	<.001	<0.001
Systolic blood pressure, mean (SD), mm Hg	130.2 (9.5)	130.4 (10.3)	129.9 (10.0)	130.2 (10.4)	.95	0.95	129.9 (9.72)	130.68 (10.2)	130.4 (10.0)	129.8 (10.3)	.61	0.68
ASCVD risk, %	7.4 (10.0)	11.3 (12.9)	14.9 (16.8)	15.3 (16.6)	<.001	<0.001	6.7 (9.2)	10.8 (13.3)	15.6 (16.8)	15.8 (16.2)	<.001	<0.001
ASCVD risk category												
Low (<5%)	176 (54.7)	136 (42.2)	133 (41.4)	130 (40.5)	<.001	<0.001	189 (58.7)	151 (46.9)	117 (36.4)	118 (36.8)	<.001	<0.001
Borderline (5%-7.5%)	26 (8.1)	23 (7.1)	14 (4.4)	10 (3.1)			24 (7.5)	16 (5.0)	19 (5.9)	14 (4.4)		
Intermediate (7.5%-20%)	89 (27.6)	99 (30.7)	70 (21.8)	73 (22.7)			76 (23.6)	94 (29.2)	87 (27.1)	74 (23.1)		
High (≥20%)	31 (9.6)	64 (19.9)	104 (32.4)	108 (33.6)			33 (10.2)	61 (18.9)	98 (30.5)	115 (35.8)		
Laboratory parameters												
HbA_1c_, mean (SD), %	6.0 (1.0)	6.3 (1.2)	6.4 (1.3)	6.6 (1.3)	<.001	<0.001	6.1 (1.1)	6.3 (1.3)	6.5 (1.3)	6.4 (1.2)	<.001	<0.001
≥7	41 (12.7)	70 (21.7)	83 (25.9)	100 (31.2)	<.001	<0.001	49 (15.2)	61 (18.9)	98 (30.5)	86 (26.8)	<.001	<0.001
Triglycerides, mean (SD), mg/dL	123.5 (65.0)	130.6 (81.5)	135.3 (82.9)	127.9 (2.0)	.25	0.32	126.7 (69.2)	128.3 (69.1)	134.6 (87.20)	127.7 (76.1)	.55	0.62
HDL, mean (SD), mg/dL	52.8 (14.1)	49.5 (14.2)	49.3 (13.6)	49.6 (13.9)	.004	0.008	52.4 (14.33)	50.7 (14.1)	49.4 (14.1)	48.7 (13.2)	.005	0.02
LDL, mean (SD), mg/dL	110.9 (32.4)	100.6 (32.6)	101.91 (33.2)	97.89 (30.5)	<.001	<0.001	107.8 (33.8)	102.0 (30.3)	101.8 (32.8)	99.7 (32.5)	.01	0.03
Coronary CTA parameters												
Coronary artery calcium score, median (IQR)	0 (0-34.7)	0 (0-60.4)	2.4 (0-139.7)	4.9 (0-186.0)	<.001	<0.001	75.9 (214.7)	123.0 (277.3)	181.4 (611.0)	217.6 (548.0)	<.001	<0.001
Coronary artery calcium score category												
0	188 (58.4)	172 (53.4)	155 (48.3)	151 (47.0)	.002	0.008	196 (60.9)	171 (53.1)	152 (47.4)	147 (45.8)	<.001	<0.001
1-10	27 (8.4)	27 (8.4)	29 (9.0)	22 (6.9)			24 (7.5)	31 (9.6)	25 (7.8)	25 (7.8)		
11-100	53 (16.5)	51 (15.8)	44 (13.7)	48 (15.0)			47 (14.6)	42 (13.0)	52 (16.2)	55 (17.1)		
101-400	35 (10.9)	48 (14.9)	51 (15.9)	49 (15.3)			36 (11.2)	44 (13.7)	58 (18.1)	45 (14.0)		
>400	19 (5.9)	24 (7.5)	42 (13.1)	51 (15.9)			19 (5.9)	34 (10.6)	34 (10.6)	49 (15.3)		
Any plaque	164 (50.9)	199 (61.8)	213 (66.4)	228 (71.0)	<.001	<0.001	162 (50.3)	195 (60.6)	221 (68.8)	226 (70.4)	<.001	<0.001
Plaque characteristics												
Calcified plaque	142 (44.1)	166 (51.6)	178 (55.5)	199 (62.0)	<.001	<0.001	137 (42.5)	164 (50.9)	189 (58.9)	195 (60.7)	<.001	<0.001
Noncalcified plaque	49 (15.2)	51 (15.8)	65 (20.2)	69 (21.5)	.10	0.17	42 (13.0)	57 (17.7)	64 (19.9)	71 (22.1)	.02	0.04
Mixed plaque	51 (15.8)	71 (22.0)	97 (30.2)	101 (31.5)	<.001	<0.001	65 (20.2)	80 (24.8)	82 (25.5)	93 (29.0)	.08	0.13
Obstructive CAD	31 (9.6)	66 (20.5)	90 (28.0)	113 (35.2)	<.001	<0.001	43 (13.4)	65 (20.2)	91 (28.3)	101 (31.5)	<.001	<0.001
No. of obstructive CAD lesions												
1 vessel	20 (6.2)	36 (11.2)	34 (10.6)	58 (18.1)	<.001	<0.001	26 (8.1)	36 (11.2)	40 (12.5)	46 (14.3)	<.001	<0.001
2 vessel	7 (2.2)	20 (6.2)	34 (10.6)	27 (8.4)			7 (2.2)	18 (5.6)	31 (9.7)	32 (10.0)		
3 vessel	4 (1.2)	10 (3.1)	21 (6.5)	28 (8.7)			9 (2.8)	11 (3.4)	20 (6.2)	23 (7.2)		
LM obstruction	2 (0.6)	3 (0.9)	9 (2.8)	9 (2.8)	.06	0.13	5 (1.6)	4 (1.2)	5 (1.6)	9 (2.8)	.45	0.62
Obstructive CAD in the LM or pLAD												
None	312 (96.9)	293 (91.0)	270 (84.1)	266 (82.9)	<.001	<0.001	307 (95.3)	293 (91.0)	275 (85.7)	266 (82.9)	<.001	<0.001
Single	9 (2.8)	27 (8.4)	43 (13.4)	47 (14.6)			12 (3.7)	25 (7.8)	42 (13.1)	47 (14.6)		
Both	1 (0.3)	2 (0.6)	8 (2.5)	8 (2.5)			3 (0.9)	4 (1.2)	4 (1.2)	8 (2.5)		
Severe CAD	8 (2.5)	17 (5.3)	49 (15.3)	46 (14.3)	<.001	<0.001	14 (4.3)	22 (6.8)	38 (11.8)	46 (14.3)	<.001	<0.001
Segment stenosis score, mean (SD)	2.8 (4.8)	4.3 (6.0)	6.3 (8.2)	7.0 (8.40)	<.001	<0.001	3.2 (5.5)	4.6 (6.7)	5.7 (7.5)	6.8 (8.2)	<.001	<0.001
Segment stenosis score ≥10	22 (6.8)	58 (18.0)	89 (27.7)	92 (28.7)	<.001	<0.001	31 (9.6)	64 (19.9)	76 (23.7)	90 (28.0)	<.001	<0.001
Segment involvement score, mean (SD)	1.7 (2.2)	2.3 (2.6)	2.912 (3.0)	3.3 (3.1)	<.001	<0.001	1.8 (2.4)	2.4 (2.8)	2.7 (2.8)	3.2 (3.0)	<.001	<0.001
Segment involvement score ≥5	28 (8.7)	44 (13.7)	68 (21.2)	80 (21.2)	<.001	<0.001	28 (8.7)	58 (18.0)	60 (18.7)	74 (23.1)	<.001	<0.001

Abbreviations: ASCVD, atherosclerotic cardiovascular disease; CAD, coronary artery disease; DCP, deep capillary plexus; FDR, false discovery rate; HbA_1c_, hemoglobin A_1c_ (glycated hemoglobin); HDL, high-density lipoprotein; LDL, low-density lipoprotein; LM, left main; NA, not applicable; OCTA, optical coherence tomography angiography; pLAD, proximal left anterior descending; SCP, superficial capillary plexus.

SI conversion factors: To convet HbA_1c_ from percentage of total hemoglobin to proportion of total hemoglobin, multiply by 0.01; triglycerides, from mg/dL to mmol/L, multiply by 0.0113; HDL and LDL, from mg/dL to mmol/L, multiply by 0.0259.

^a^ Multiple comparisons were adjusted using the Benjamini-Hochberg procedure to control the FDR.

### Incremental Prognostic Value of Retinal PFVD

For a detailed evaluation of the clinical usefulness of PFVD in the diagnosis of subclinical CAD, AUC values were compared (Table 3; eTable 9 in Supplement 1). Models with SCP PFVD alone, DCP PFVD alone, and both SCP and DCP PFVD showed incremental benefits over traditional cardiovascular risk factors for identifying severe CAD, obstructive CAD, and SSS of 10 or higher. Notably, adding SCP PFVD tended to yield better diagnostic performance for severe CAD, obstructive CAD, and SSS of 10 or higher compared to DCP PFVD. The ROC curves and AUC values for subclinical CAD diagnosis are depicted in eFigures 8 and 9 in Supplement 1. Addition of PFVD in cardiovascular risk factors showed high negative predictive value (NPV, >0.9) for obstructive CAD and severe CAD and high positive predictive value (PPV, >0.9) for elevated SSS and SIS scores. The logistic training and random forest models incorporating OCTA parameters alongside traditional cardiovascular risk factors demonstrated good discriminative performance, particularly for severe and obstructive CAD, with the random forest models achieving higher sensitivity and specificity (eFigures 8-10 and eTable 10 in Supplement 1).

**Table 3.** Incremental Value of Optical Coherence Tomography Angiography Variables Over Clinical Risk Factors for Diagnosing Subclinical Coronary Atherosclerosis

Model	AUC (95% CI)	*P* value for comparison	Sensitivity	Specificity	PPV	NPV
**Severe CAD**						
Cardiovascular risk factors^a^	0.78 (0.74-0.81)	Reference	0.78	0.68	0.20	0.97
Cardiovascular risk factors + SCP PFVD^a^	0.78 (0.75-0.82)	.05	0.71	0.76	0.23	0.96
Cardiovascular risk factors + DCP PFVD^a^	0.78 (0.75-0.82)	.12	0.84	0.62	0.19	0.98
Cardiovascular risk factors + SCP PFVD + DCP PFVD^a^	0.79 (0.75-0.82)^b^	.03^b^	0.81	0.65	0.19	0.97
**Obstructive CAD**						
Cardiovascular risk factors^a^	0.77 (0.74-0.80)	Reference	0.84	0.58	0.38	0.92
Cardiovascular risk factors + SCP PFVD^a^	0.78 (0.75-0.80)^b^	.01^b^	0.86	0.58	0.38	0.93
Cardiovascular risk factors + DCP PFVD^a^	0.77 (0.75-0.80)	.07	0.81	0.64	0.40	0.92
Cardiovascular risk factors + SCP PFVD + DCP PFVD^a^	0.78 (0.75-0.81)^b^	.01^b^	0.85	0.60	0.39	0.93
**Any plaque**						
Cardiovascular risk factors^a^	0.74 (0.71-0.76)	Reference	0.65	0.71	0.79	0.55
Cardiovascular risk factors + SCP PFVD^a^	0.74 (0.71-0.76)	.51	0.68	0.67	0.78	0.56
Cardiovascular risk factors^a^ + DCP PFVD	0.74 (0.71-0.76)	.44	0.68	0.67	0.78	0.56
Cardiovascular risk factors^a^ + SCP PFVD + DCP PFVD	0.74 (0.71-0.76)	.61	0.80	0.56	0.75	0.62
**CACS >400**						
Cardiovascular risk factors^a^	0.75 (0.71-0.79)	Reference	0.82	0.59	0.19	0.96
Cardiovascular risk factors^a^ + SCP PFVD	0.75 (0.72-0.79)	.09	0.84	0.57	0.19	0.97
Cardiovascular risk factors^a^ + DCP PFVD	0.69 (0.72-0.80)	.11	0.78	0.64	0.20	0.96
Cardiovascular risk factors^a^ + SCP PFVD + DCP PFVD	0.76 (0.72-0.80)	.10	0.78	0.64	0.20	0.96
**SSS ≥10**						
Cardiovascular risk factors^a^	0.76 (0.72-0.79)	Reference	0.58	0.34	0.93	0.16
Cardiovascular risk factors + SCP PFVD^a^	0.77 (0.74-0.80)^b^	.005^b^	0.70	0.38	0.91	0.22
Cardiovascular risk factors + DCP PFVD^a^	0.76 (0.73-0.79)^b^	.04^b^	0.59	0.34	0.94	0.16
Cardiovascular risk factors + SCP PFVD + DCP PFVD^a^	0.77 (0.74-0.78)^b^	.005^b^	0.57	0.34	0.94	0.15
**SIS ≥5**						
Cardiovascular risk factors^a^	0.75 (0.72-0.79)	Reference	0.70	0.33	0.92	0.19
Cardiovascular risk factors + SCP PFVD^a^	0.76 (0.72-0.79)	.36	0.69	0.33	0.92	0.19
Cardiovascular risk factors + DCP PFVD^a^	0.76 (0.72-0.79)	.44	0.60	0.29	0.94	0.14
Cardiovascular risk factors + SCP PFVD + DCP PFVD^a^	0.76 (0.72-0.79)	.30	0.70	0.33	0.92	0.19

Abbreviations: AUC, area under the curve; CACS, coronary artery calcium score; CAD, coronary artery disease; DCP, deep capillary plexus; NPV, negative predictive value; PFVD, parafoveal vascular density; PPV, positive predictive value; SCP, superficial capillary plexus; SIS, segment involvement score; SSS, segment stenosis score.

^a^ Cardiovascular risk factor: traditional risk factor − age, sex, smoking, hypertension, diabetes, hyperlipidemia, body mass index category.

^b^ Significant values.

### Cutoff Value of OCTA Parameters for Diagnosing Risk of Subclinical CAD

The optimal cutoff values of PFVD for diagnosing obstructive and severe CAD were 46.3% for SCP and 49.6% for DCP (eFigures 11 and 12 in Supplement 1); values below these thresholds were defined as low PFVD for subsequent analyses (eFigure 13 and eTable 11 in Supplement 1). Consistent with previous results in the study, participants with low SCP PFVD showed an increased likelihood of severe CAD (adjusted OR [aOR], 3.13; 95% CI, 1.93-5.24) and obstructive CAD (aOR, 1.89; 95% CI, 1.39-2.57). Similar but weaker associations were observed for low DCP PFVD, but strong associations for those with combined low SCP and DCP PFVD. A sensitivity analysis in the subgroup of patients aged 30 to 79 years, adjusting for PREVENT risk score, showed consistent results (eFigure 14 in Supplement 1).

**Figure 2.**
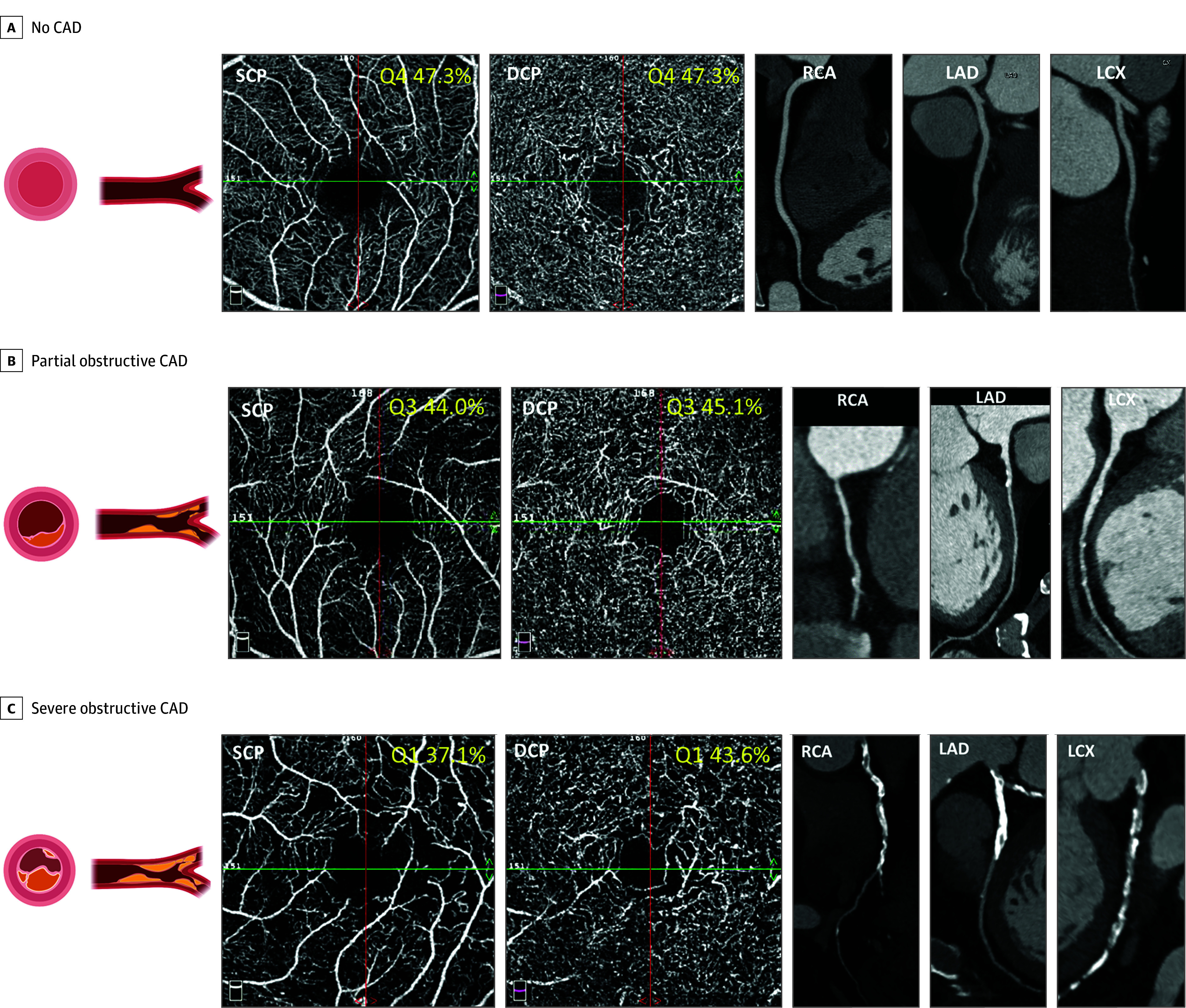
Schematic Summary and Representative Cases of Retinal Parafoveal Vascular Density and Coronary Computed Tomography Angiography No coronary artery disease (CAD) (A), partial obstructive CAD (B), and severe obstructive CAD (C). DCP indicates deep capillary plexus; LAD, left anterior descending coronary artery; LCX, left circumflex artery; RCA, right coronary artery; SCP, superficial capillary plexus; Q, quartile.

Additionally, we conducted sensitivity analyses restricted to participants with diabetes in the study cohort. While the overall trends remained consistent, the strength of the associations was attenuated, likely due to the reduced sample size in this subgroup (eFigures 15 and 16 and eTables 12-16 in Supplement 1). Figure 2 illustrates representative cases showing an association between OCTA and coronary CTA.

## Discussion

This cross-sectional cohort study demonstrates that retinal microvasculature, particularly PFVD, is closely associated with systemic comorbidities and serves as a potential indicator of subclinical coronary atherosclerosis. A decrease in PFVD is associated with an increased likelihood of composite subclinical atherosclerosis findings in coronary CTA in a dose-dependent manner. Among the evaluated parameters, SCP PFVD emerged as the most sensitive marker associated with coronary atherosclerosis, and low SCP PFVD could be a supportive tool for diagnosing the disease.

Our findings reveal that among the retinal microvascular parameters measured by OCTA, PFVD exhibits the strongest correlation with atherosclerotic burden as determined by coronary CTA. Furthermore, SCP and DCP PFVD significantly improved the AUC values for identifying coronary atherosclerosis risk (particularly obstructive CAD and severe CAD) beyond traditional CAD risk factors. This aligns with previous research by Drinkwater and colleagues,^12^ which demonstrated a significant association between carotid stenosis and lower PFVD in patients with type 2 diabetes. Since there is a normal variation of FAZ among individuals, PFVD, which might be less affected by FAZ size, could better represent retinal microvascular status compared to other OCTA metrics.^27,28^ Recent work by Ren and colleagues^29^ using OCTA showed impaired retinal microcirculation in patients with nonobstructive and obstructive CAD, suggesting that even early coronary atherosclerosis may manifest in the retinal microvasculature. Our study builds upon these findings by demonstrating that reduced PFVD correlates not only with nonobstructive but also obstructive CAD and overall disease burden as quantified by SSS and SIS in a larger population and comparative risk modeling. Our findings add to the growing evidence supporting the utility of impaired retinal microvascular structure as a valuable biomarker for assessing CAD risk.

This study demonstrated that low PFVD was closely associated with severe CAD and obstructive CAD. This association is important, as severe and obstructive CAD are linked to increased mortality and major cardiac events.^3^ Similarly, Cheung and colleagues^30^ reported an independent association between retinal arteriolar narrowing and left ventricular hypertrophy in healthy individuals. Furthermore, Wong and colleagues^31^ demonstrated that retinopathy predicted incident heart failure over 7 years among healthy individuals. Moreover, previous population-based studies, such as ARIC, MESA, and SEED, have shown that retinal vascular changes (eg, retinal vascular abnormalities, narrowing of retinal vessel caliber) are associated with increased cardiovascular risk.^32,33,34,35,36^ Compared to these findings, our OCTA-based assessment of parafoveal capillary density offers a more detailed and quantitative approach. The magnitude of association we observed between OCTA parameters and subclinical CAD is consistent with previously reported correlations using fundus photography–based parameters. Currently, while coronary screening is frequently considered for patients with known CVDs, such as diabetes, it is commonly overlooked in individuals without known systemic diseases.^37^ In this regard, OCTA has the potential to serve as a supportive tool for identifying individuals who may benefit from coronary examination, such as coronary CTA. Our findings suggest that reduced PFVD may warrant closer cardiovascular risk assessment, even in otherwise healthy, asymptomatic individuals.

The addition of retinal PFVD to traditional cardiovascular risk factors modestly improved the diagnosis of subclinical coronary atherosclerosis, with an AUC approaching 0.8. While the incremental gain in discriminative power was limited, the clinical value lies in the potential application of OCTA as a noninvasive, opportunistic screening tool in real-world ophthalmology settings. The prevalence of obstructive CAD was higher than in our previous asymptomatic cohorts, likely reflecting the older age and greater cardiometabolic burden of the current participants.^38,39,40^ Given that individuals with diabetes or other systemic vascular risk factors may be more likely to attend ophthalmic screenings, OCTA could serve as an opportunistic window to identify those at risk for subclinical CVD. PFVD demonstrated outcome-dependent diagnostic utility: while high NPV supported its role in ruling out severe or obstructive CAD, its strong PPV for higher SSS and SIS scores suggests potential for identifying individuals with a greater subclinical atherosclerotic burden. These findings support the role of OCTA in augmenting cardiovascular risk stratification where access to coronary calcium scoring or CTA is limited.

Notably, SCP PFVD was found to sensitively reflect systemic comorbidities and was significantly associated with patients’ coronary artery status. Consistent with these findings, prior studies support that lower SCP VD is significantly associated with the cardiovascular profile of patients experiencing acute coronary syndrome and coronary total occlusion.^9,11^ SCP contains relatively larger retinal vessels and more arteries and arterioles compared with DCP, which mostly contains capillaries, veins, and venules.^4^ Previous research has also demonstrated that molecular regulation of the blood-retinal barrier of the SCP and DCP is different, and signs of retinal arterial dysfunction are preferentially shown in the SCP compared with DCP.^4^ Given the previously observed association between coronary arterial endothelial dysfunction and peripheral arterial endothelial dysfunction, it is plausible that the autoregulatory function of retinal arteries and arterioles in the SCP may also be compromised in patients with CAD.^11,41^ Therefore, systemic microvascular dysfunction might be sensitively represented as decreased VD in the retinal SCP parafoveal area.

### Limitations

This study has several limitations. First, as a retrospective, single-center study in a single-ethnicity, clinic-based cohort, generalizability may be limited. Second, advanced bilateral macular diseases (eg, diabetic macular edema) were excluded due to poor image quality, which may have reduced statistical power but was necessary to minimize outliers and ensure analytic robustness. Third, as the study cohort consisted of asymptomatic individuals with a high burden of cardiometabolic risk factors, the findings may not be directly generalizable to the general population. However, given that individuals with diabetes or other systemic vascular risk factors may be more likely to attend ophthalmic screenings, OCTA could serve as an opportunistic window to identify those at risk for subclinical CVD. Fourth, axial length data were not available in our dataset. However, all participants underwent thorough retinal examination by experts to exclude high myopia. Since the prescence of borderline high myopia could possibly interfere with OCTA measurements, future studies incorporating axial length measurements are warranted to clarify this issue. Fifth, coronary plaque characteristics were not assessed. As plaque composition may provide prognostic insight beyond stenosis severity, this warrants further investigation. Lastly, our analysis did not differentiate between retinal arteries, veins, and capillaries. The use of artificial intelligence–assisted analysis of the different types of retinal vessels in the future could potentially clarify this issue.

## Conclusions

In conclusion, this large clinic-based cohort study provides evidence that retinal microvasculature measurements by OCTA are associated with subclinical coronary atherosclerosis. When integrated with conventional cardiovascular risk factors, reduced PFVD may serve as a complementary marker to help identify asymptomatic individuals who could benefit from further cardiovascular evaluation.

## Notes

Supplement 1. eMethods. Supplementary Methods and References eFigure 1. Flowchart of the Study eFigure 2. Correlation and Scatter Plots Show the Relationship Between Optical Coherence Tomography Angiography (OCTA) Parameters and Coronary Computed Tomography Angiography (CTA) Parameters eFigure 3. Pearson Matrix and Relevant Variance Inflation Factor (VIF) of Continuous Variables of Optical Coherence Tomography Angiography (OCTA) Parameters and Coronary Computed Tomography Angiography (CTA) Parameters eFigure 4. Visualization of Linearity and Correlation Using Scatterplots With Fitted Linear and LOESS Curves eFigure 5. Logistic Regression Models Using SCP and DCP PFVD as Continuous Predictors (per Unit Decrease) eFigure 6. Distribution of PFVD Stratified by Subclinical Coronary Atherosclerosis Outcomes eFigure 7. Association Between Retinal Parafoveal Vascular Density and Subclinical Coronary Atherosclerosis in Participants Age 30-79 eFigure 8. Incremental Prognostic Value of Parafoveal Vascular Density for Diagnosing Subclinical Coronary Atherosclerosis (Severe CAD, Obstructive CAD, and CACS> 400) eFigure 9. Incremental Prognostic Value of Parafoveal Vascular Density for Diagnosing Subclinical Coronary Atherosclerosis (Any Plaque, SSS≥ 10, SIS≥ 5) eFigure 10. Prognostic Value of Retinal Parafoveal Vascular Density for Subclinical Coronary Atherosclerosis: A Model Validation With Random Forest eFigure 11. Sensitivity and Specificity Analysis of Retinal Parafoveal VD Cutoff for Predicting Severe CAD eFigure 12. Sensitivity and Specificity Analysis of Retinal Parafoveal VD Cutoff for Predicting Obstructive CAD eFigure 13. Association of Low Parafoveal Vascular Density With Subclinical Coronary Atherosclerosis eFigure 14. Association of Low Parafoveal Vascular Density With Subclinical Coronary Atherosclerosis in Participants Age 30-79 eFigure 15. Association Between Retinal Parafoveal Vascular Density and Subclinical Coronary Atherosclerosis in Participants With Diabetes eFigure 16. Incremental Prognostic Value of Parafoveal Vascular Density for Diagnosing Subclinical Coronary Atherosclerosis in Patients With Diabetes eTable 1. Relevant Ophthalmic Condition for OCTA Examination eTable 2. Clinical Characteristics of the Participants (Complete Case vs. Missing Case) eTable 3. Pearson and Spearman Correlation Coefficients Between OCTA Parameters and Coronary Atherosclerosis Measures eTable 4. Quartile Presentation of Clinical Characteristics eTable 5. Logistic Regression Analysis for Parafoveal Vascular Density and CAD Outcomes eTable 6. Odds Ratios per Unit Increase in Parafoveal Vascular Density for Coronary Atherosclerosis Outcomes eTable 7. Variance Inflation Factor (VIF) Values of Factors in Logestic Regression Model eTable 8. Clinical Characteristics of the Participants Age 30-79 eTable 9. Comparative AUC Analysis of SCP and DCP Parafoveal Vascular Density (PFVD) in Identifying Subclinical Coronary Atherosclerosis eTable 10. Diagnostic Performance Metrics of Validation Results With Random Forest eTable 11. Odds Ratios for Low SCP and DCP PFVD in the Full Cohort and in Patients Aged 30–79 Years eTable 12. Clinical Characteristics of the Participants Stratified by Diabetes eTable 13. Coronary Computed Tomography Angiography Parameters by Retinal Parafoveal Vascular Density Quartile in Diabetic Patients eTable 14. Logistic Regression Analysis for Parafoveal Vascular Density and CAD Outcomes in the DM Subgroup eTable 15. Incremental Value of Optical Coherence Tomography Angiography Variables Over Clinical Risk Factors for Diagnosing Subclinical Coronary Atherosclerosis in Patients With Diabetes eAppendix. STROBE Checklist

Supplement 2. Data Sharing Statement
