# Supplementary material for: Subclinical Coronary Atherosclerosis and Retinal Optical Coherence Tomography Angiography
Source: JAMA Cardiol. 2025 Sep 17;10(11):1100–11. doi: 10.1001/jamacardio.2025.3036 (PMC12444650; doi:10.1001/jamacardio.2025.3036)
Supplement: Supplement 1. — eMethods. Supplementary Methods and References eFigure 1. Flowchart of the Study eFigure 2. Correlation and Scatter Plots Show the Relationship Between Optical Coherence Tomography Angiography (OCTA) Parameters and Coronary Computed Tomography Angiography (CTA) Parameters eFigure 3. Pearson Matrix and Relevant Variance Inflation Factor (VIF) of Continuous Variables of Optical Coherence Tomography Angiography (OCTA) Parameters and Coronary Computed Tomography Angiography (CTA) Parameters eFigure 4. Visualization of Linearity and Correlation Using Scatterplots With Fitted Linear and LOESS Curves eFigure 5. Logistic Regression Models Using SCP and DCP PFVD as Continuous Predictors (per Unit Decrease) eFigure 6. Distribution of PFVD Stratified by Subclinical Coronary Atherosclerosis Outcomes eFigure 7. Association Between Retinal Parafoveal Vascular Density and Subclinical Coronary Atherosclerosis in Participants Age 30-79 eFigure 8. Incremental Prognostic Value of Parafoveal Vascular Density for Diagnosing Subclinical Coronary Atherosclerosis (Severe CAD, Obstructive CAD, and CACS> 400) eFigure 9. Incremental Prognostic Value of Parafoveal Vascular Density for Diagnosing Subclinical Coronary Atherosclerosis (Any Plaque, SSS≥ 10, SIS≥ 5) eFigure 10. Prognostic Value of Retinal Parafoveal Vascular Density for Subclinical Coronary Atherosclerosis: A Model Validation With Random Forest eFigure 11. Sensitivity and Specificity Analysis of Retinal Parafoveal VD Cutoff for Predicting Severe CAD eFigure 12. Sensitivity and Specificity Analysis of Retinal Parafoveal VD Cutoff for Predicting Obstructive CAD eFigure 13. Association of Low Parafoveal Vascular Density With Subclinical Coronary Atherosclerosis eFigure 14. Association of Low Parafoveal Vascular Density With Subclinical Coronary Atherosclerosis in Participants Age 30-79 eFigure 15. Association Between Retinal Parafoveal Vascular Density and Subclinical Coronary Atherosclerosis in Participants With Diabetes eFigure 16. [file jamacardiol-e253036-s001.pdf]

Supplemental Online Content

Yang JM, Yang DH, Lee SW, et al. Subclinical coronary atherosclerosis and retinal optical coherence tomography angiography. *JAMA Cardiol*. Published online September 17, 2025. doi:10.1001/jamacardio.2025.3036

eMethods. Supplementary Methods and References

eFigure 1. Flowchart of the Study

eFigure 2. Correlation and Scatter Plots Show the Relationship Between Optical Coherence Tomography Angiography (OCTA) Parameters and Coronary Computed Tomography Angiography (CTA) Parameters

eFigure 3. Pearson Matrix and Relevant Variance Inflation Factor (VIF) of Continuous Variables of Optical Coherence Tomography Angiography (OCTA) Parameters and Coronary Computed Tomography Angiography (CTA) Parameters

eFigure 4. Visualization of Linearity and Correlation Using Scatterplots With Fitted Linear and LOESS Curves

eFigure 5. Logistic Regression Models Using SCP and DCP PFVD as Continuous Predictors (per Unit Decrease)

eFigure 6. Distribution of PFVD Stratified by Subclinical Coronary Atherosclerosis Outcomes

eFigure 7. Association Between Retinal Parafoveal Vascular Density and Subclinical Coronary Atherosclerosis in Participants Age 30-79

eFigure 8. Incremental Prognostic Value of Parafoveal Vascular Density for Diagnosing Subclinical Coronary Atherosclerosis (Severe CAD, Obstructive CAD, and CACS> 400)

eFigure 9. Incremental Prognostic Value of Parafoveal Vascular Density for Diagnosing Subclinical Coronary Atherosclerosis (Any Plaque, SSS≥ 10, SIS≥ 5)

eFigure 10. Prognostic Value of Retinal Parafoveal Vascular Density for Subclinical Coronary Atherosclerosis: A Model Validation With Random Forest

eFigure 11. Sensitivity and Specificity Analysis of Retinal Parafoveal VD Cutoff for Predicting Severe CAD

eFigure 12. Sensitivity and Specificity Analysis of Retinal Parafoveal VD Cutoff for Predicting Obstructive CAD

eFigure 13. Association of Low Parafoveal Vascular Density With Subclinical Coronary Atherosclerosis

eFigure 14. Association of Low Parafoveal Vascular Density With Subclinical Coronary Atherosclerosis in Participants Age 30-79

eFigure 15. Association Between Retinal Parafoveal Vascular Density and Subclinical Coronary Atherosclerosis in Participants With Diabetes

eFigure 16. Incremental Prognostic Value of Parafoveal Vascular Density for Diagnosing Subclinical Coronary Atherosclerosis in Patients With Diabetes

© 2025 Yang JM et al. *JAMA Cardiology.*

eTable 1. Relevant Ophthalmic Condition for OCTA Examination

eTable 2. Clinical Characteristics of the Participants (Complete Case vs. Missing Case)

eTable 3. Pearson and Spearman Correlation Coefficients Between OCTA Parameters and Coronary Atherosclerosis Measures

eTable 4. Quartile Presentation of Clinical Characteristics

eTable 5. Logistic Regression Analysis for Parafoveal Vascular Density and CAD Outcomes

eTable 6. Odds Ratios per Unit Increase in Parafoveal Vascular Density for Coronary Atherosclerosis Outcomes

eTable 7. Variance Inflation Factor (VIF) Values of Factors in Logestic Regression Model

eTable 8. Clinical Characteristics of the Participants Age 30-79

eTable 9. Comparative AUC Analysis of SCP and DCP Parafoveal Vascular Density (PFVD) in Identifying Subclinical Coronary Atherosclerosis

eTable 10. Diagnostic Performance Metrics of Validation Results With Random Forest

eTable 11. Odds Ratios for Low SCP and DCP PFVD in the Full Cohort and in Patients Aged 30–79 Years

eTable 12. Clinical Characteristics of the Participants Stratified by Diabetes

eTable 13. Coronary Computed Tomography Angiography Parameters by Retinal Parafoveal Vascular Density Quartile in Diabetic Patients

eTable 14. Logistic Regression Analysis for Parafoveal Vascular Density and CAD Outcomes in the DM Subgroup

eTable 15. Incremental Value of Optical Coherence Tomography Angiography Variables Over Clinical Risk Factors for Diagnosing Subclinical Coronary Atherosclerosis in Patients With Diabetes

eAppendix. STROBE Checklist

This supplemental material has been provided by the authors to give readers additional information about their work.

© 2025 Yang JM et al. *JAMA Cardiology*.

eMethods. Supplementary Methods and References

Coronary CTA image acquisition and analysis (detailed version)

All participants underwent multidetector coronary CTA using a second or third-generation dual-source scanner (Somatom Definition, Siemens, Germany) following standard scanning protocol, as previously described [1,2]. All coronary CTA images were interpreted by three board-certified radiologists based on the established interpretation guidelines, and a cardiovascular radiologist (YDH) summarized the results based on their interpretations.[3] The standard coronary artery tree model was applied based on the Society of Cardiovascular Computed Tomography guidelines.[4] Coronary artery calcium score (CACS) was measured and categorized as previously reported.[1] Plaques were classified as calcified (density >130 Hounsfield units in native scans, with >50% calcified tissue), mixed (50% calcium), or noncalcified (no calcium). At the site of maximum stenosis, the contrast-enhanced part of the coronary channel was traced semi-automatically and compared to the mean value of the proximal and distal reference sites. Obstructive CAD with significant stenosis was defined as stenosis ≥ 50%. The total atherosclerotic plaque burden was assessed using a coronary artery plaque score: segment involvement score (SIS) and segment stenosis score (SSS). Severe CAD was defined as meeting one of the following criteria: (1) ≥2-vessel coronary disease involving the proximal left anterior descending coronary artery (LAD) involvement, (2) 3-vessel disease, or (3) left main coronary artery (LM) disease [5].

Sample size calculation

Sample size estimation was conducted using PASS 15 software (NCSS, Kaysville, UT, USA). Assuming an expected odds ratio of 1.5, a two-sided α of 0.05, and 80% power, a minimum of 1,087 participants was required. Our final cohort of 1,286 exceeded this threshold, ensuring sufficient power for detecting clinically meaningful associations in multivariable models.

Diagnostic Threshold Determination and cut-off values

© 2025 Yang JM et al. *JAMA Cardiology.*

The optimal cutoff value for SCP and DCP PFVD, as well as for other ROC-based models were identified using Youden's index, which determines the threshold that maximizes the sum of sensitivity and specificity. These cut-offs were then used to calculate the corresponding diagnostic performance metrics including sensitivity, specificity, positive predictive value (PPV) and negative predictive value (NPV). The OCTA variables were divided into quartiles (lowest VD [quartile 1] to highest VD [quartile 4]) and the baseline characteristics, and coronary CTA variables were compared. The risk of subclinical atherosclerosis was assessed for each quartile. Dichotomization of continuous outcome variables (CACS, SSS, and SIS) were based on clinically accepted thresholds to facilitate interpretability and clinical relevance.

Predictive modeling for validation of diagnostic value of OCTA parameter incorporation

To validate the diagnostic utility of incorporating OCTA parameters, we constructed binary classification models by training logistic regression and random forest algorithms. The dataset was randomly partitioned into a training set (80%) and a test set (20%) using stratified sampling based on the outcome variable.

Within the training data, 10-fold cross-validation was applied to optimize model parameters and assess internal validity. Final model performance was evaluated on the test set using the area under the receiver operating characteristic curve (AUC), accuracy, sensitivity, specificity, positive predictive value (PPV), and negative predictive value (NPV). Random forest models were implemented using 500 trees, with variable importance calculated by impurity-based metrics.

Statistical analysis

The optimal cutoff value for SCP and DCP PFVD, as well as for other ROC-based models were identified using Youden's index, which determines the threshold that maximizes the sum of sensitivity and specificity. These cut-offs were then used to calculate the corresponding diagnostic performance

© 2025 Yang JM et al. *JAMA Cardiology*.

metrics including sensitivity, specificity, positive predictive value (PPV) and negative predictive value (NPV). Logistic regression models were used to calculate odds ratios (ORs) with 95% confidence intervals (CIs) adjusted for confounders as follows: (1) model 1 (minimally adjusted): age, sex; (2) model 2: adjusted for traditional CAD risk factors (age, male sex, hypertension, diabetes, hyperlipidemia, history of smoking, body mass index categories (<25, 25–30, ≥30), atherosclerotic cardiovascular disease (ASCVD) risk [6,7]

Supplementary references

1. Kang SH, Park GM, Lee SW, et al. Long-Term Prognostic Value of Coronary CT Angiography in Asymptomatic Type 2 Diabetes Mellitus. *JACC Cardiovasc Imaging*. 2016;9(11):1292-1300. doi:10.1016/j.jcmg.2016.01.040

2. Lee SB, Park GM, Lee JY, et al. Association between non-alcoholic fatty liver disease and subclinical coronary atherosclerosis: An observational cohort study. *J Hepatol*. 2018;68(5):1018-1024. doi:10.1016/j.jhep.2017.12.012

3. Kim C, Park CH, Lee BY, et al. 2024 Consensus Statement on Coronary Stenosis and Plaque Evaluation in CT Angiography From the Asian Society of Cardiovascular Imaging-Practical Tutorial (ASCI-PT). *Korean J Radiol*. 2024;25(4):331-342. doi:10.3348/kjr.2024.0112

4. Hecht HS, Cronin P, Blaha MJ, et al. 2016 SCCT/STR guidelines for coronary artery calcium scoring of noncontrast noncardiac chest CT scans: A report of the Society of Cardiovascular Computed Tomography and Society of Thoracic Radiology. *J Cardiovasc Comput Tomogr*. 2017;11(1):74-84. doi:10.1016/j.jcct.2016.11.003

5. Min JK, Berman DS, Dunning A, et al. All-cause mortality benefit of coronary revascularization vs. medical therapy in patients without known coronary artery disease undergoing coronary computed tomographic angiography: Results from CONFIRM (COronary CT Angiography EvaluatioN for Clinical Out. *Eur Heart J*. 2012;33(24):3088-

© 2025 Yang JM et al. *JAMA Cardiology.*

3097. doi:10.1093/eurheartj/ehs315

6. Del Rincón I, Williams K, Stern MP, Freeman GL, Escalante A. High incidence of cardiovascular events in a rheumatoid arthritis cohort not explained by traditional cardiac risk factors. *Arthritis Rheum*. 2001;44(12):2737-2745. doi:10.1002/1529-0131(200112)44:12<2737::aid-art460>3.0.co;2-%23

7. Ajufo E, Ayers CR, Vigen R, et al. Value of coronary artery calcium scanning in association with the net benefit of aspirin in primary prevention of atherosclerotic cardiovascular disease. *JAMA Cardiol*. 2021;6(2):179-187. doi:10.1001/jamacardio.2020.4939

© 2025 Yang JM et al. *JAMA Cardiology*.

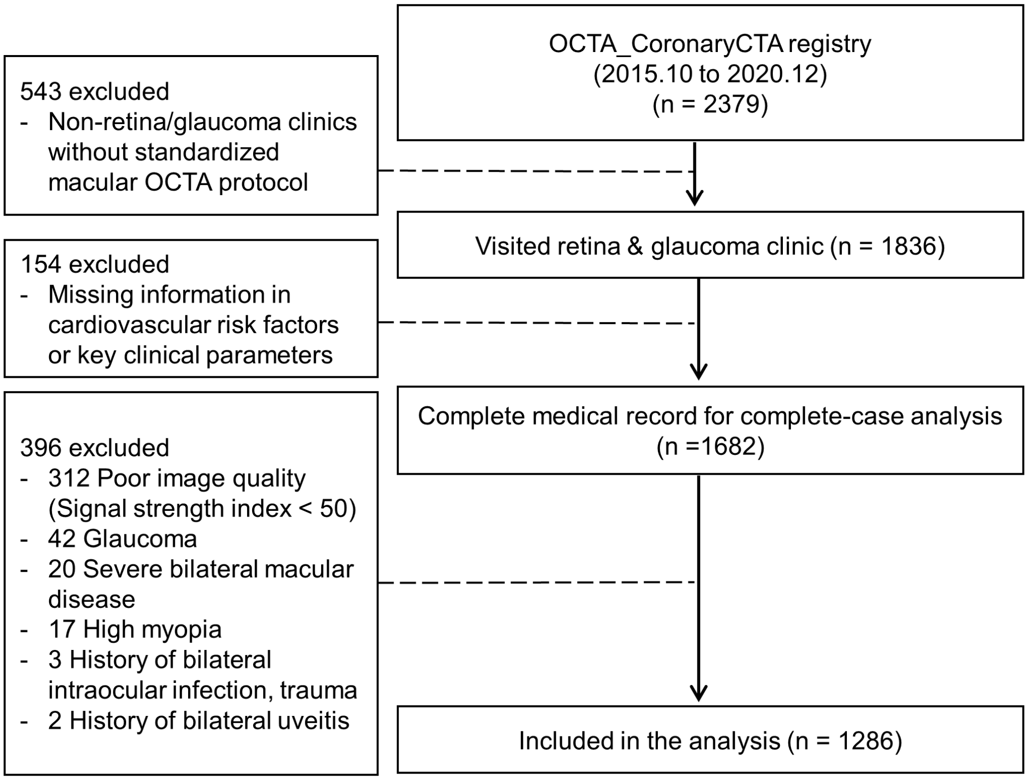

eFigure 1. Flowchart of the Study

© 2025 Yang JM et al. *JAMA Cardiology*.

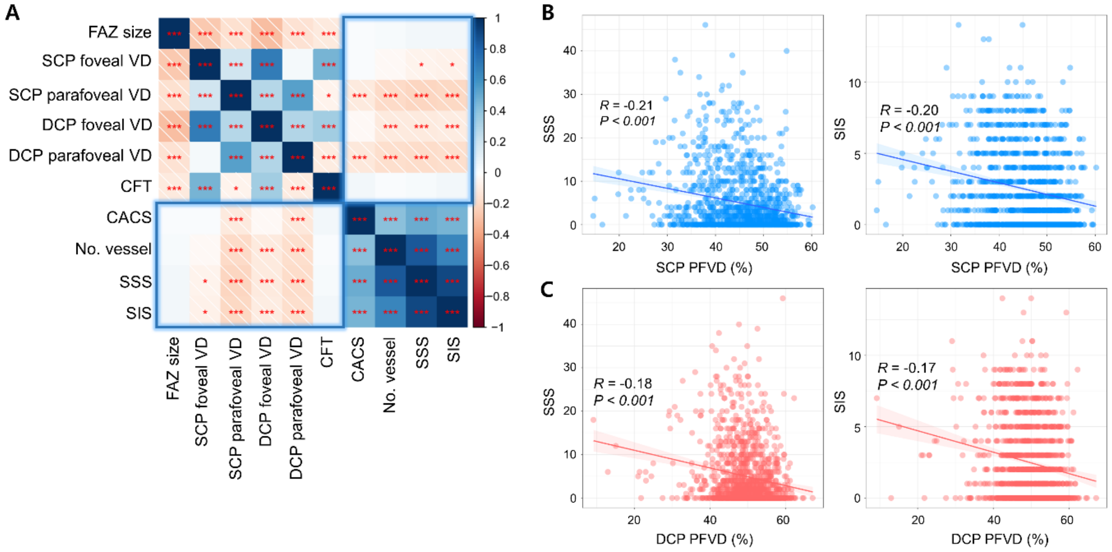

eFigure 2. Correlation and Scatter Plots Show the Relationship Between Optical Coherence Tomography Angiography (OCTA) Parameters and Coronary Computed Tomography Angiography (Cta) Parameters

(A) Correlation matrix plot. * P<0.05, ** P<0.01, *** P<0.001. (B) Scatter plot with regression lines between segment stenosis score (SSS), segment involvement score (SIS), and superficial capillary plexus (SCP) parafoveal vascular density (PFVD). (C) Scatter plot with regression lines between SSS, SIS, and deep capillary plexus (DCP) PFVD.

Abbreviations: CACS, Coronary artery calcium score; CFT, central foveal thickness, DCP, deep capillary plexus parafoveal vascular density; SCP; superficial capillary plexus; SIS, segment involvement score; SSS, segment stenosis score; VD, vascular density

© 2025 Yang JM et al. *JAMA Cardiology.*

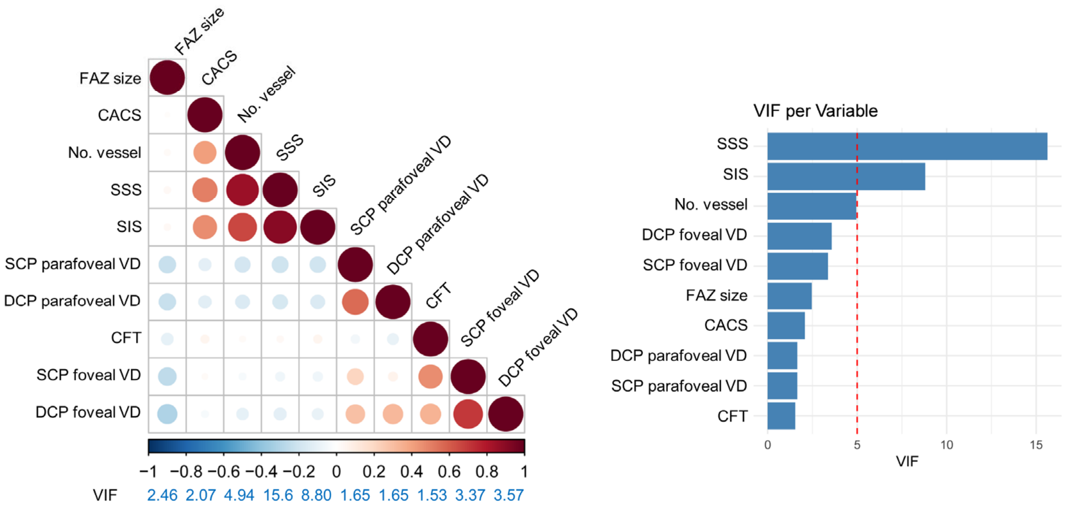

eFigure 3. Pearson Matrix and Relevant Variance Inflation Factor (VIF) of Continuous Variables of Optical Coherence Tomography Angiography (Octa) Parameters and Coronary Computed Tomography Angiography (Cta) Parameters

Abbreviations: CACS, Coronary artery calcium score; CFT, central foveal thickness, DCP, deep capillary plexus parafoveal vascular density; SCP; superficial capillary plexus; SIS, segment involvement score; SSS, segment stenosis score; VIF, variance inflation factor, VD, vascular density

© 2025 Yang JM et al. *JAMA Cardiology.*

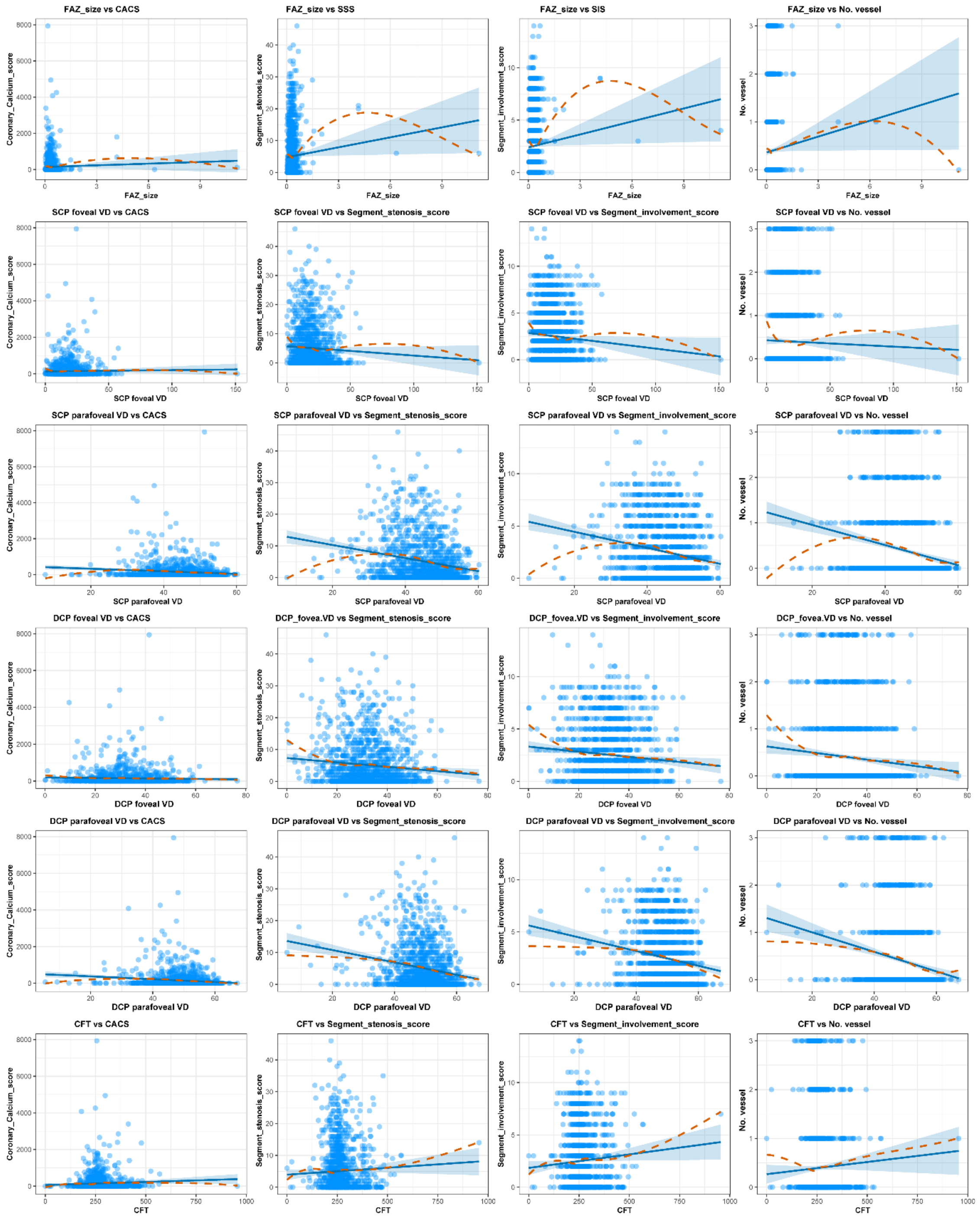

eFigure 4. Visualization of Linearity and Correlation Using Scatterplots With Fitted Linear and LOESS Curves

© 2025 Yang JM et al. *JAMA Cardiology.*

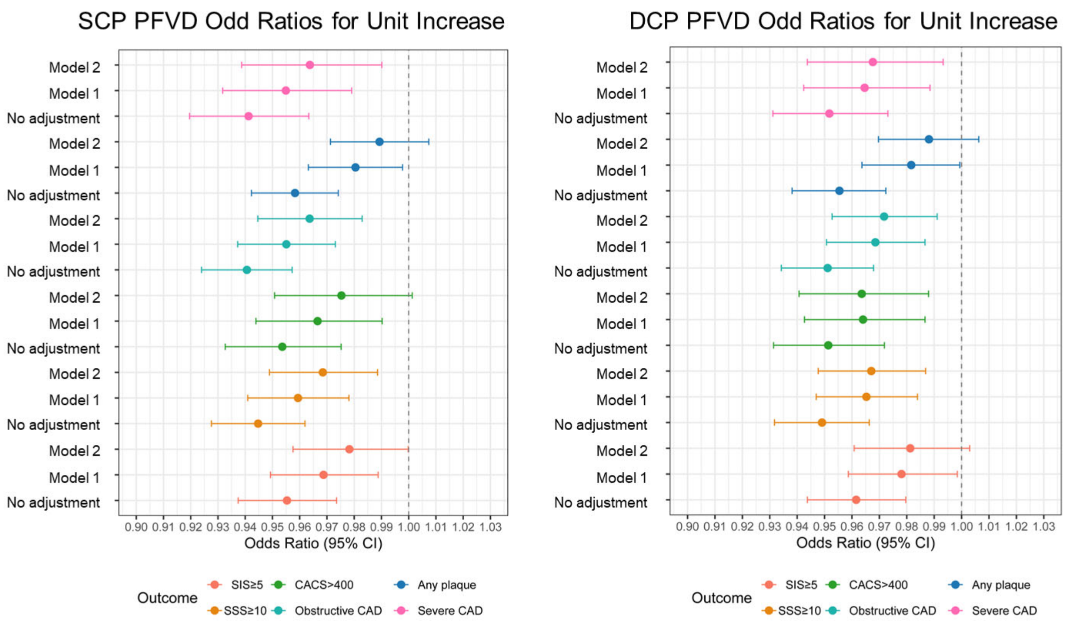

eFigure 5. Logistic Regression Models Using SCP and DCP PFVD as Continuous Predictors (per Unit Decrease)

© 2025 Yang JM et al. *JAMA Cardiology*.

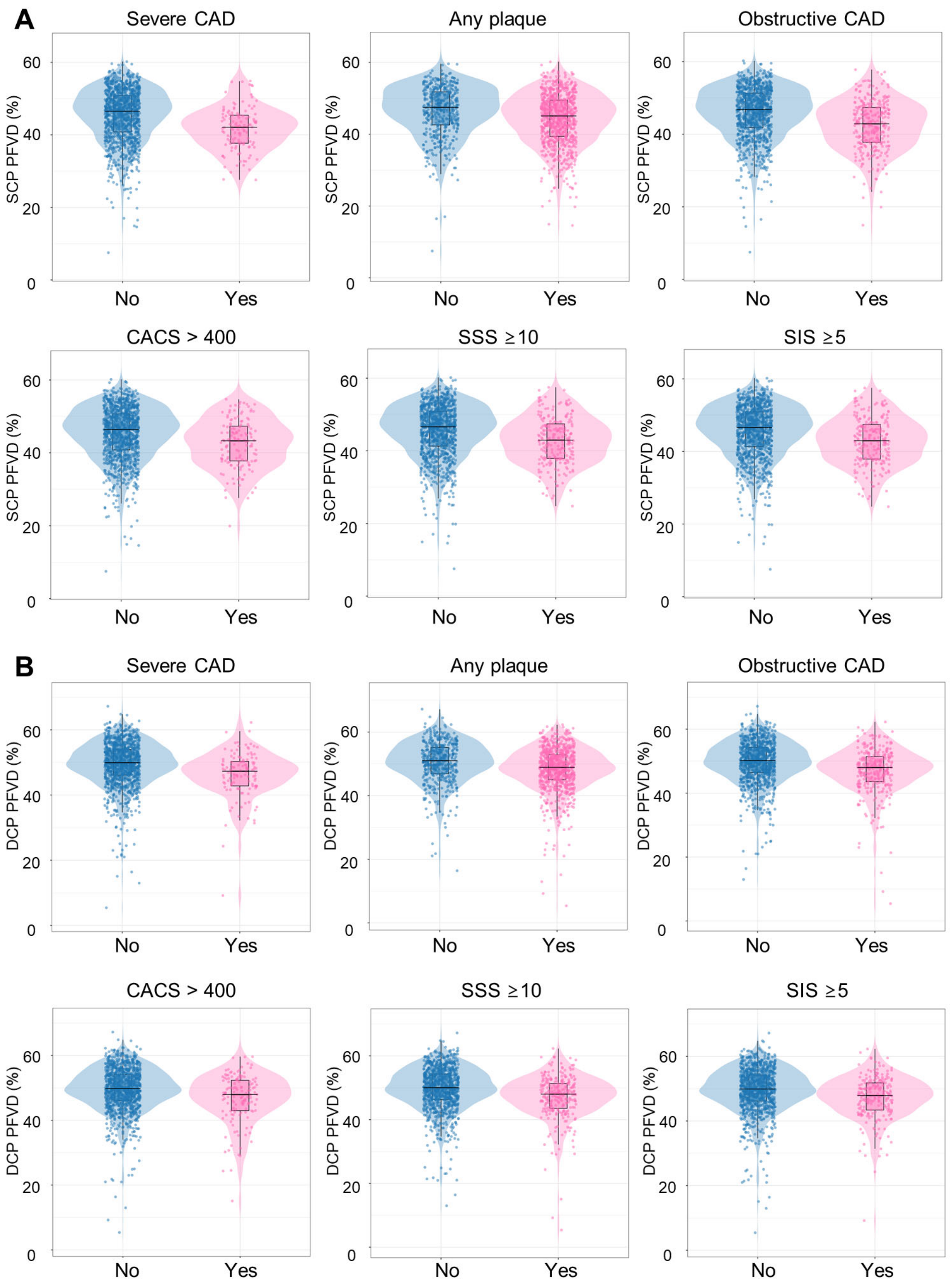

eFigure 6. Distribution of PFVD Stratified by Subclinical Coronary Atherosclerosis Outcomes

(A) Superficial capillary plexus (SCP) PFVD. **(B)** Deep capillary plexus (DCP) PFVD.

© 2025 Yang JM et al. *JAMA Cardiology.*

Abbreviations: CAD, coronary artery disease; CFT, central foveal thickness, DCP, deep capillary plexus parafoveal vascular density; SIS, segment involvement score; SSS, segment stenosis score

© 2025 Yang JM et al. *JAMA Cardiology*.

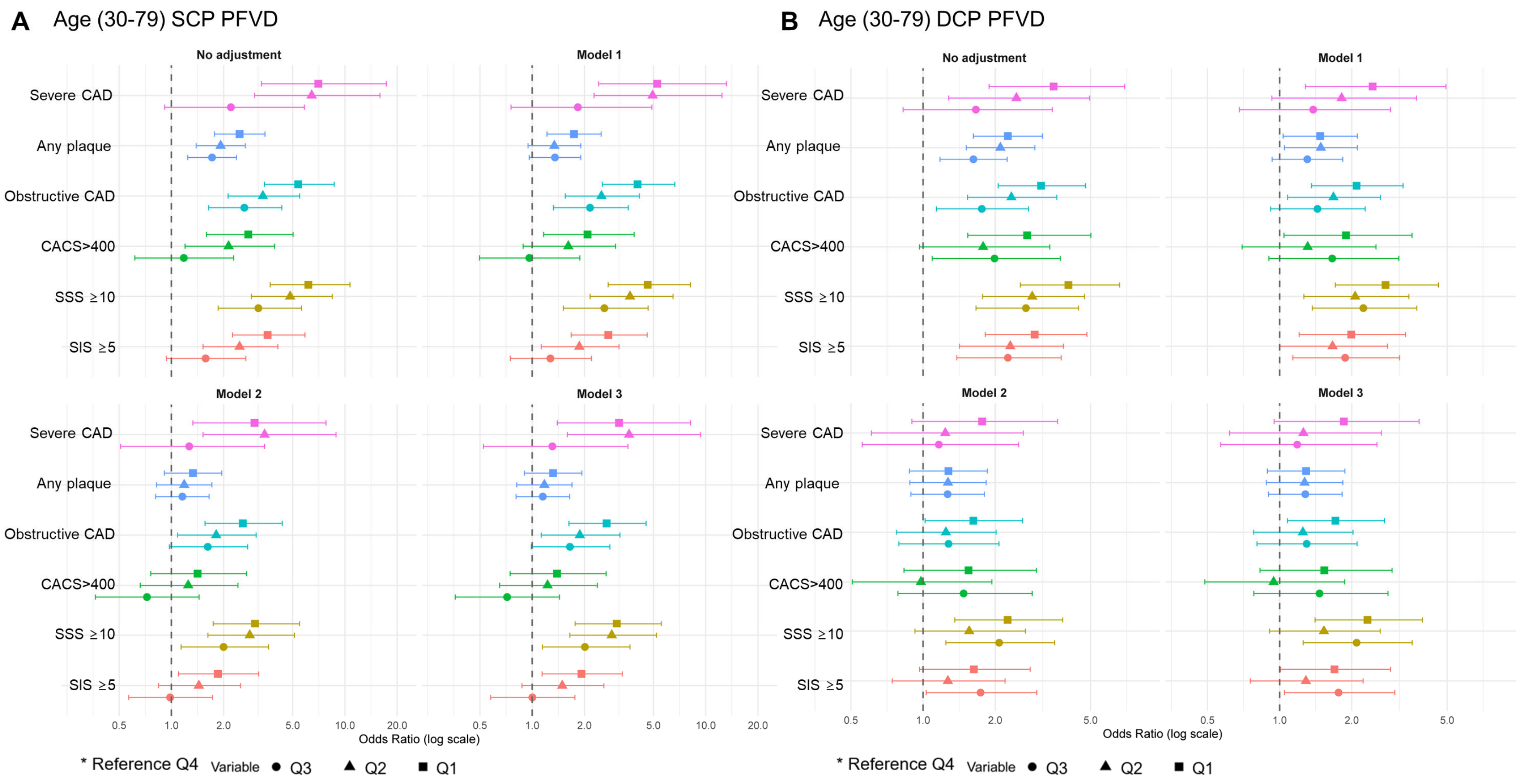

eFigure 7. Association Between Retinal Parafoveal Vascular Density and Subclinical Coronary Atherosclerosis in Participants Age 30-79

(A) Forest plot of logestic regression analyses using quartile of superficial capillary plexus (SCP) parafoveal vascular density (PFVD) and (B) Deep capillary plexus (SCP) PFVD. Reference: Q4 (highest quartile), Model 1: Adjusted for age and sex, Model 2: Adjusted for age, sex, traditional cardiovascular risk factors (hypertension, diabetes, hyperlipidemia, history of smoking, and body mass index categories), and PREVENT score, Model 3: Adjusted for age, sex,

© 2025 Yang JM et al. *JAMA Cardiology*.

traditional cardiovascular risk factors (hypertension, diabetes, hyperlipidemia, history of smoking, and body mass index categories), and PREVENT score category.

CAD, coronary artery disease; CACS, coronary artery calcium score; SIS, segment involvement score; SSS, segment stenosis score

© 2025 Yang JM et al. *JAMA Cardiology*.

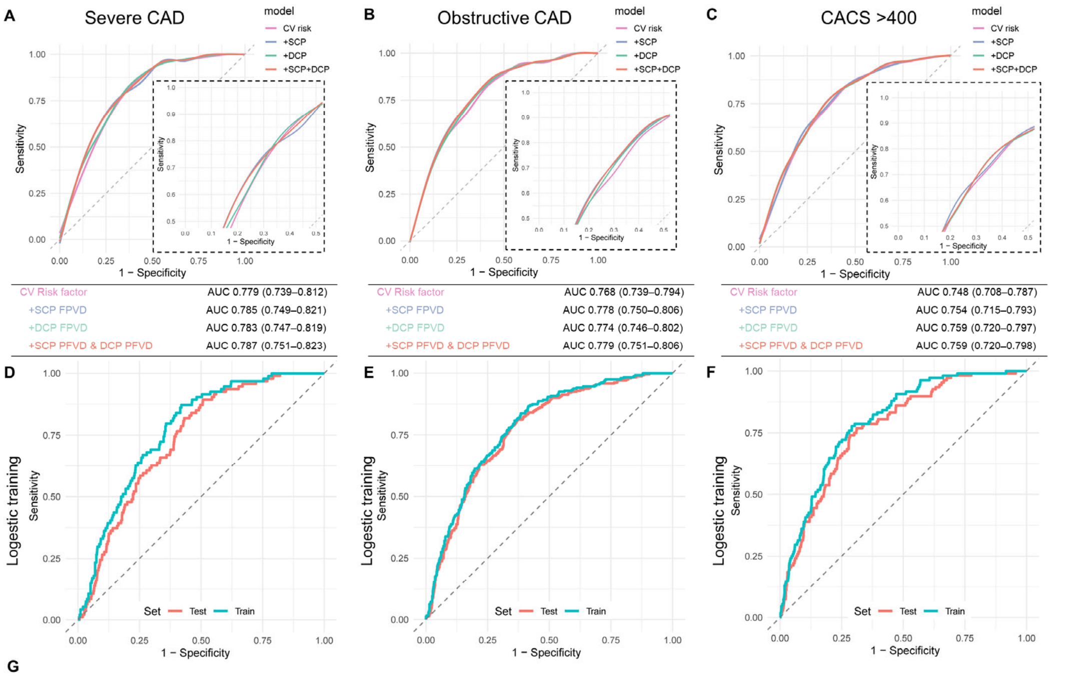

G

Set	AUC	Sens	Spec	PPV	NPV	Acc
Train	0.833 (0.775-0.891)	0.839	0.531	0.946	0.254	0.810
Test	0.800 (0.760-0.840)	0.803	0.640	0.954	0.258	0.787

Set	AUC	Sens	Spec	PPV	NPV	Acc
Train	0.767 (0.704-0.830)	0.816	0.588	0.867	0.493	0.763
Test	0.765 (0.733-0.798)	0.813	0.550	0.856	0.471	0.752

Set	AUC	Sens	Spec	PPV	NPV	Acc
Train	0.734 (0.716-0.788)	0.666	0.708	0.949	0.207	0.670
Test	0.689 (0.592-0.786)	0.474	0.821	0.956	0.160	0.512

eFigure 8. Incremental Prognostic Value of Parafoveal Vascular Density for Diagnosing Subclinical Coronary Atherosclerosis (Severe CAD, Obstructive CAD, and CACS> 400)

(A–C) Receiver operating characteristic (ROC) curve for diagnosing subclinical coronary atherosclerosis. Inset panel: enlarged view. **(D–G)** Logistic prediction models using 10-fold cross validation. AUC, area under the curve; Acc, accuracy; CAD, coronary artery disease; DCP, deep capillary plexus

© 2025 Yang JM et al. *JAMA Cardiology.*

parafoveal vascular density; NPV, negative predictive value; PPV, positive predictive value; SCP, superficial capillary plexus; SIS, segment involvement score; SSS, segment stenosis score

© 2025 Yang JM et al. *JAMA Cardiology*.

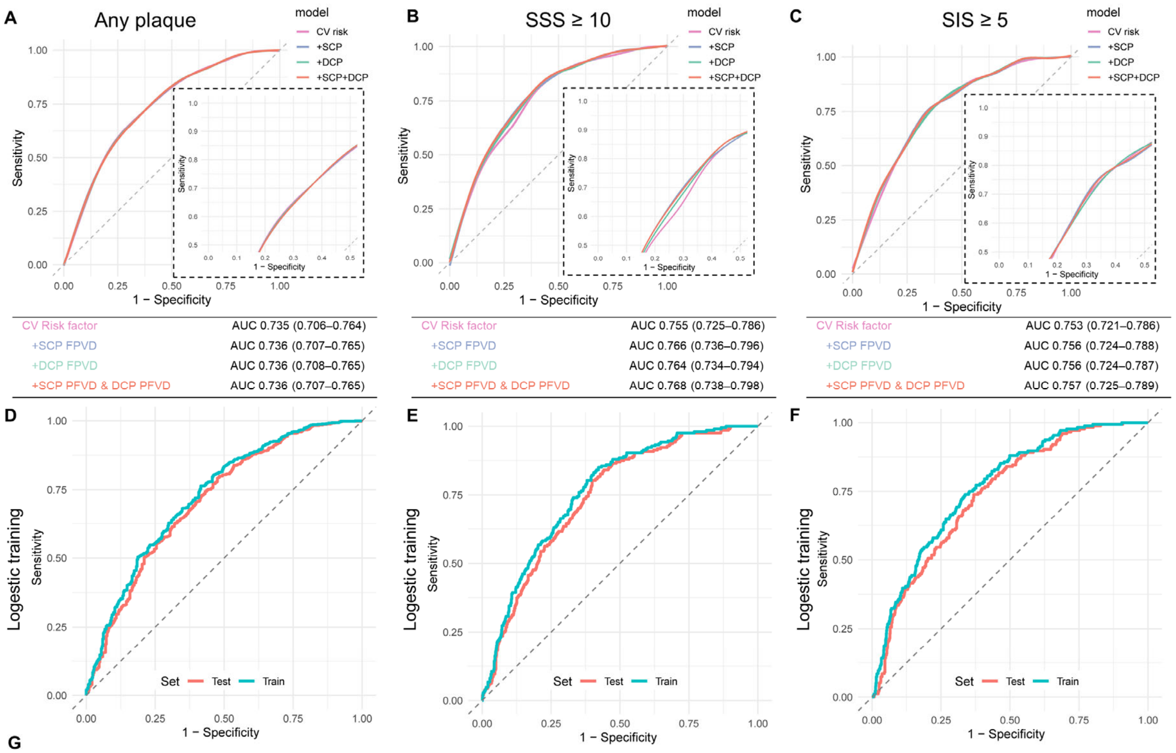

G

Set	AUC	Sens	Spec	PPV	NPV	Acc
Train	0.710 (0.677-0.743)	0.579	0.777	0.615	0.750	0.701
Test	0.707 (0.640-0.774)	0.691	0.701	0.570	0.799	0.698

Set	AUC	Sens	Spec	PPV	NPV	Acc
Train	0.752 (0.716-0.788)	0.780	0.623	0.887	0.428	0.747
Test	0.731 (0.758-0.804)	0.537	0.895	0.947	0.354	0.616

Set	AUC	Sens	Spec	PPV	NPV	Acc
Train	0.735 (0.697-0.773)	0.759	0.602	0.899	0.348	0.732
Test	0.659 (0.575-0.744)	0.495	0.818	0.930	0.250	0.550

eFigure 9. Incremental Prognostic Value of Parafoveal Vascular Density for Diagnosing Subclinical Coronary Atherosclerosis (Any Plaque, SSS≥ 10, SIS≥ 5)

© 2025 Yang JM et al. *JAMA Cardiology*.

(A–C) Receiver operating characteristic (ROC) curve for diagnosing subclinical coronary atherosclerosis. Inset panel: enlarged view. **(D–G)** Logistic prediction models using 10-fold cross validation. AUC, area under the curve; Acc, accuracy; CAD, coronary artery disease; DCP, deep capillary plexus parafoveal vascular density; NPV, negative predictive value; PPV, positive predictive value; SCP, superficial capillary plexus; SIS, segment involvement score; SSS, segment stenosis score

© 2025 Yang JM et al. *JAMA Cardiology*.

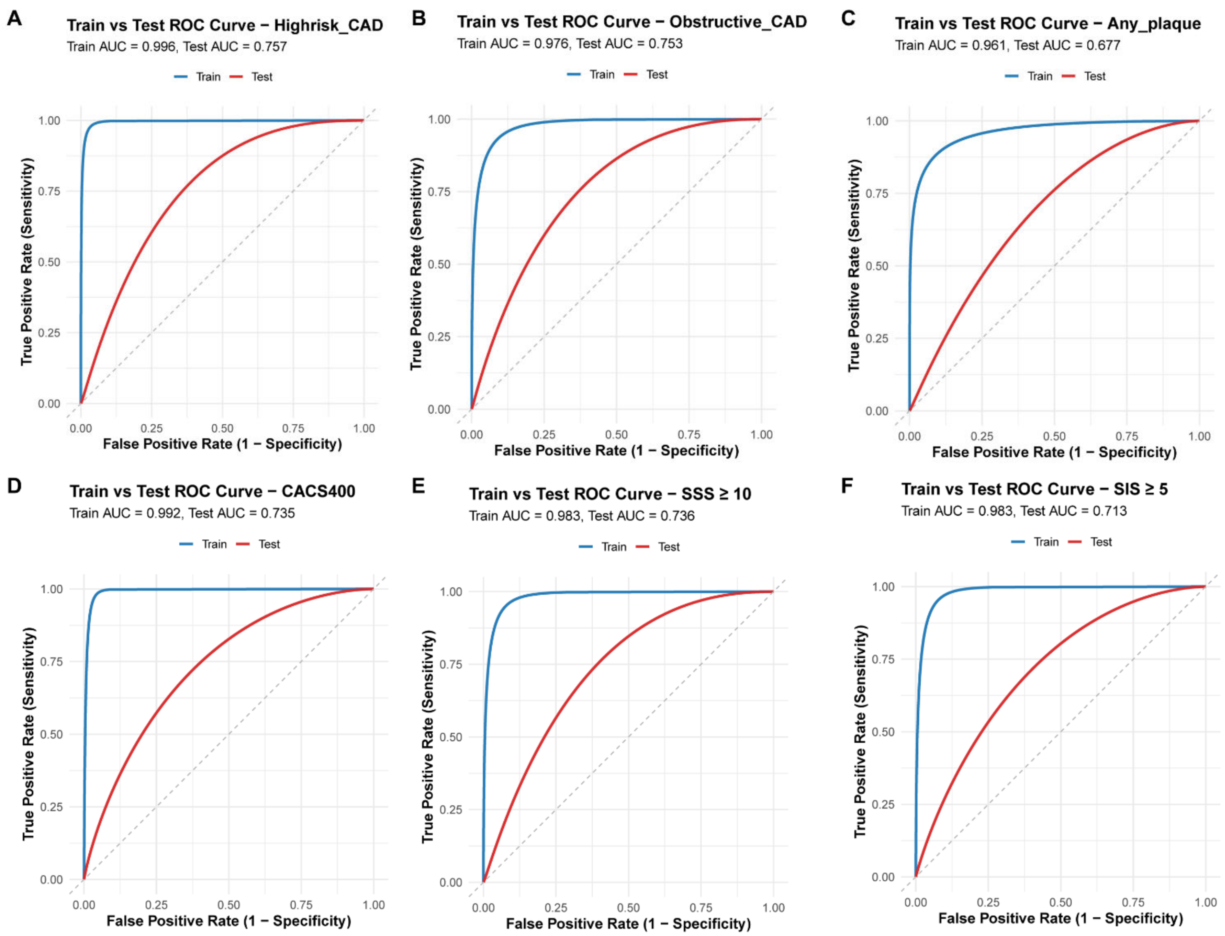

eFigure 10. Prognostic Value of Retinal Parafoveal Vascular Density for Subclinical Coronary Atherosclerosis: A Model Validation With Random Forest

© 2025 Yang JM et al. *JAMA Cardiology*.

Cut-off value severe CAD

SCP

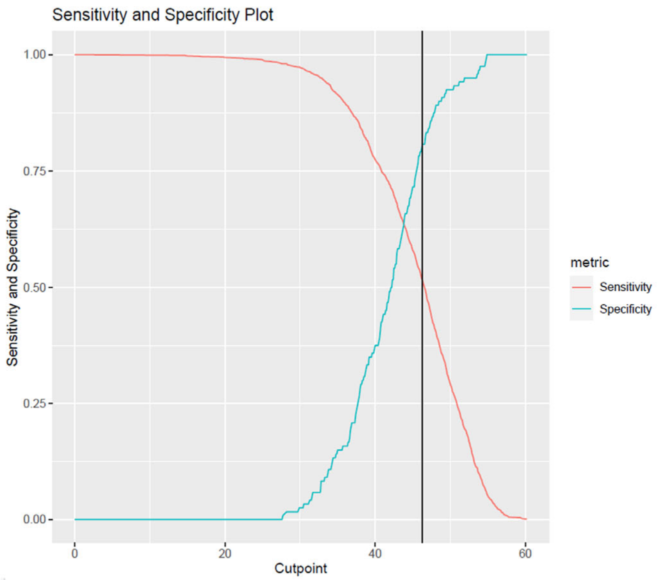

AUC	n	n_pos	n_neg
0.6763	1286	1166	120

optimal_cutpoint	sum_sens_spec	acc	sensitivity	specificity	tp	fn	fp	tn
46.3	1.3246	0.5435	0.5163	0.8083	602	564	23	97

Predictor summary:

Data	Min.	5%	1st Qu.	Median	Mean	3rd Qu.	95%	Max.	SD	NAs
Overall	0.0	32.625	40.500	46.0	45.00420	50.400	54.800	60.2	7.315118	0
0	0.0	32.825	40.800	46.5	45.35009	50.800	55.000	60.2	7.361588	0
1	27.6	31.595	37.675	42.1	41.64333	45.425	51.885	54.8	5.892464	0

DCP

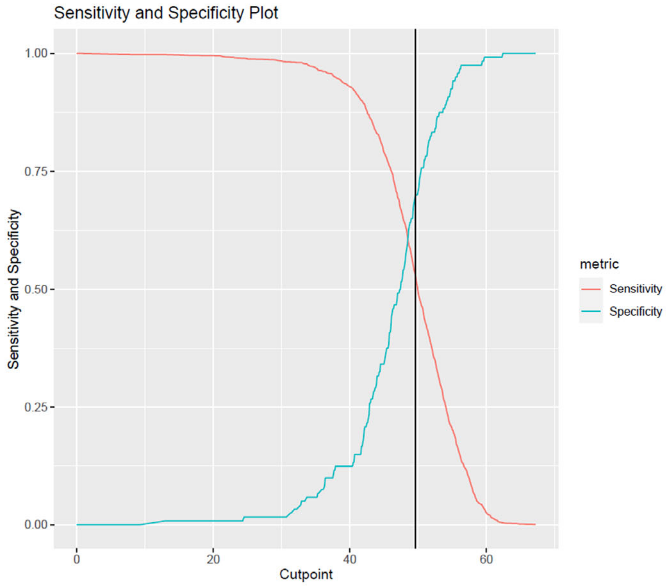

AUC	n	n_pos	n_neg
0.6443	1286	1166	120

optimal_cutpoint	sum_sens_spec	acc	sensitivity	specificity	tp	fn	fp	tn
49.6	1.2309	0.5467	0.5309	0.7	619	547	36	84

Predictor summary:

Data	Min.	5%	1st Qu.	Median	Mean	3rd Qu.	95%	Max.	SD	NAs
Overall	0.0	37.400	45.7	49.7	49.08095	53.700	58.500	67.2	7.164955	0
0	0.0	37.825	46.1	49.9	49.37539	54.000	58.575	67.2	7.098771	0
1	9.2	33.370	42.8	47.3	46.22000	50.325	55.415	62.3	7.204627	0

eFigure 11. Sensitivity and Specificity Analysis of Retinal Parafoveal VD Cutoff for Predicting Severe CAD

© 2025 Yang JM et al. *JAMA Cardiology.*

Cut-off value obstructive CAD

SCP

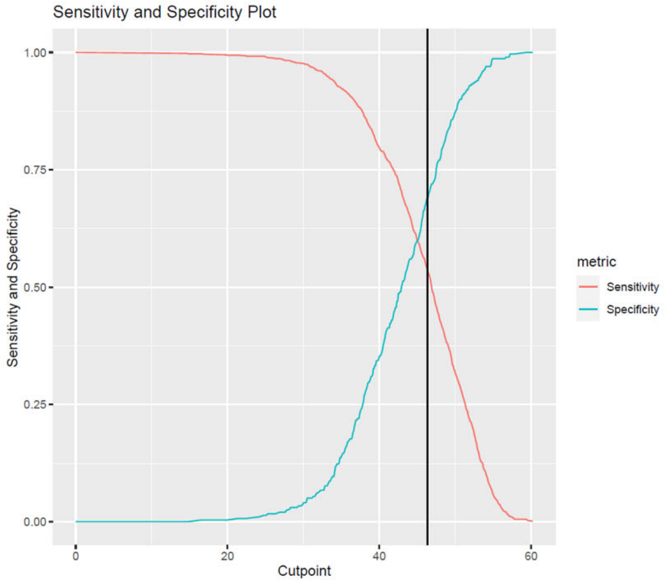


```
   AUC    n n_pos n_neg
0.6483 1286   986   300
```

```
optimal_cutpoint sum_sens_spec    acc sensitivity specificity  tp  fn fp  tn
            46.3        1.2296 0.5747      0.5396        0.69 532 454 93 207
```

```
Predictor summary:
    Data Min.     5% 1st Qu. Median     Mean 3rd Qu.    95% Max.       SD NAS
 Overall  0.0 32.625  40.500  46.00 45.00420  50.400 54.800 60.2 7.315118   0
       0  0.0 33.050  41.800  46.80 45.79777  51.275 55.175 60.2 7.245151   0
       1 14.9 31.065  37.775  42.85 42.39600  47.400 53.205 57.8 6.938573   0
```

DCP

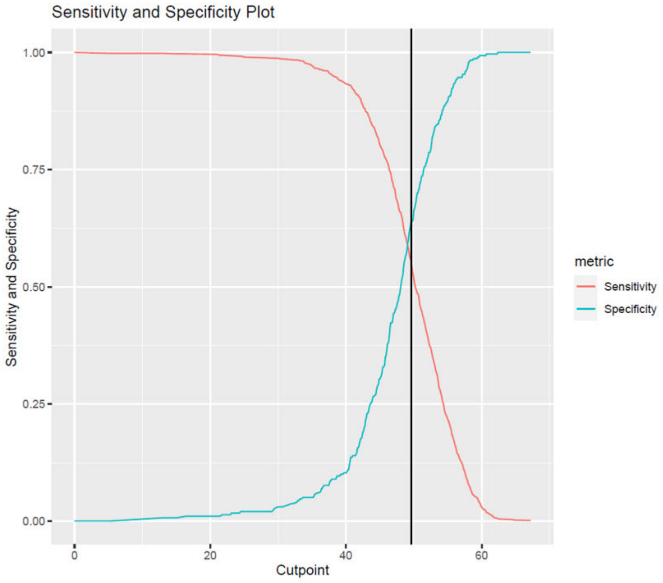


```
   AUC    n n_pos n_neg
0.6213 1286   986   300
```

```
optimal_cutpoint sum_sens_spec    acc sensitivity specificity  tp  fn  fp  tn
            49.6        1.1948 0.5747      0.5548        0.64 547 439 108 192
```

```
Predictor summary:
    Data Min.     5% 1st Qu. Median     Mean 3rd Qu.  95% Max.       SD NAS
 Overall  0.0 37.400  45.700   49.7 49.08095    53.7 58.5 67.2 7.164955   0
       0  0.0 38.125  46.300   50.2 49.72434    54.3 58.9 67.2 6.959505   0
       1  5.4 35.110  43.575   48.0 46.96633    51.4 57.1 62.3 7.430766   0
>
```

eFigure 12. Sensitivity and Specificity Analysis of Retinal Parafoveal VD Cutoff for Predicting Obstructive CAD

© 2025 Yang JM et al. *JAMA Cardiology.*

Variables	OR (95% CI)		
	No adjustment	Model 1	Model 2
**Low SCP PFVD (<46.3%)**			
Severe CAD	4.50 (2.87-7.36)	3.63 (2.27-6.01)	3.13(1.93-5.24)
Obstructive CAD	2.61(1.99-3.44)	2.95(1.57-2.81)	1.89(1.39-2.57)
**Low DCP PFVD (<49.6%)**			
Severe CAD	2.64(1.77-4.01)	2.03(1.34-3.12)	1.75(1.14-2.72)
Obstructive CAD	2.22(1.70-2.90)	1.68(1.27-2.23)	1.51(1.13-2.03)
**Low SCP & DCP PFVD**			
Severe CAD	3.59(2.44-5.33)	2.87(1.93-4.32)	2.53(1.67-3.86)
Obstructive CAD	2.39(1.83-3.11)	1.89(1.43-2.50)	1.74(1.30-2.33)

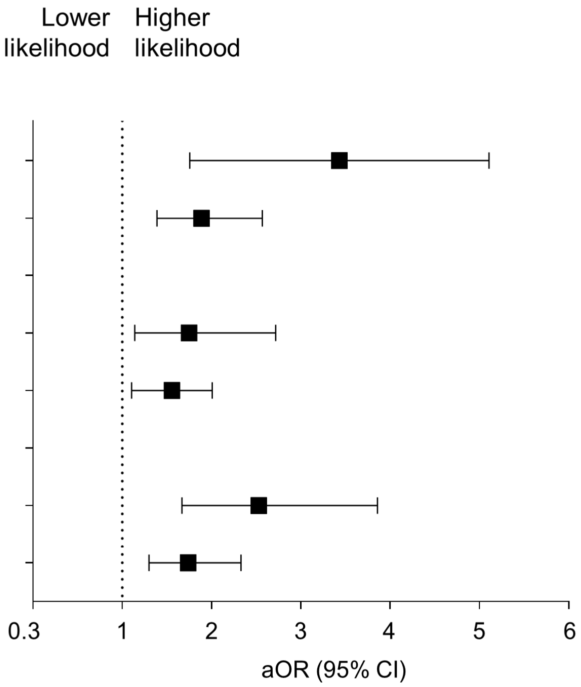

eFigure 13. Association of Low Parafoveal Vascular Density With Subclinical Coronary Atherosclerosis

Model 1: Adjusted for age and sex, Model 2: Adjusted for age, sex, and traditional cardiovascular risk factors (hypertension, diabetes, hyperlipidemia, history of smoking, and body mass index categories), and atherosclerotic cardiovascular disease (ASCVD) risk

Abbreviations: aOR, adjusted odd ratio; CAD, coronary artery disease; CI, confidence interval; DCP, deep capillary plexus; OR, odd ratio; PFVD, parafoveal vascular density; SCP, superficial capillary plexus

© 2025 Yang JM et al. *JAMA Cardiology.*

	OR (95% CI)			
**Variables**	**No adjustment**	**Model 1**	**Model 2**	**Model 3**
**Low SCP PFVD (<46.3%)**				
Severe CAD	4.27(2.68-7.09)	3.53(2.18-5.95)	2.78(1.67-4.76)	2.85(1.72-4.90)
Obstructive CAD	2.47(1.87-3.30)	2.03(1.51-2.74)	1.62(1.17-2.24)	1.67(1.21-2.30)
**Low DCP PFVD (<49.6%)**				
Severe CAD	2.47(1.63-3.82)	1.94(1.26-3.05)	1.51(0.96-2.42)	1.56(0.99-2.49)
Obstructive CAD	2.19(1.68-2.90)	1.68(1.26-2.26)	1.37(1.00-1.88)	1.41(1.03-1.93)
**Low SCP & DCP PFVD**				
Severe CAD	3.26(2.17-4.93)	2.67(1.75-4.09)	2.14(1.37-3.34)	2.21(1.42-3.45)
Obstructive CAD	2.29(1.73-3.02)	1.85(1.38-2.47)	1.51(1.10-2.07)	1.57(1.15-2.15)

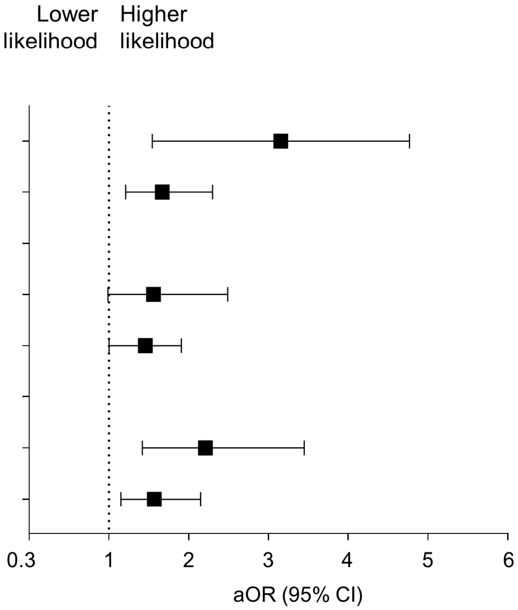

eFigure 14. Association of Low Parafoveal Vascular Density With Subclinical Coronary Atherosclerosis in Participants Age 30-79

Reference: Q4 (highest quartile), Model 1: Adjusted for age and sex, Model 2: Adjusted for age, sex, traditional cardiovascular risk factors (hypertension, diabetes, hyperlipidemia, history of smoking, and body mass index categories), and PREVENT score, Model 3: Adjusted for age, sex, traditional cardiovascular risk factors (hypertension, diabetes, hyperlipidemia, history of smoking, and body mass index categories), and PREVENT score category.

Abbreviations: aOR, adjusted odd ratio; CAD, coronary artery disease; CI, confidence interval; DCP, deep capillary plexus; OR, odd ratio; PFVD, parafoveal vascular density; SCP, superficial capillary plexus

© 2025 Yang JM et al. *JAMA Cardiology.*

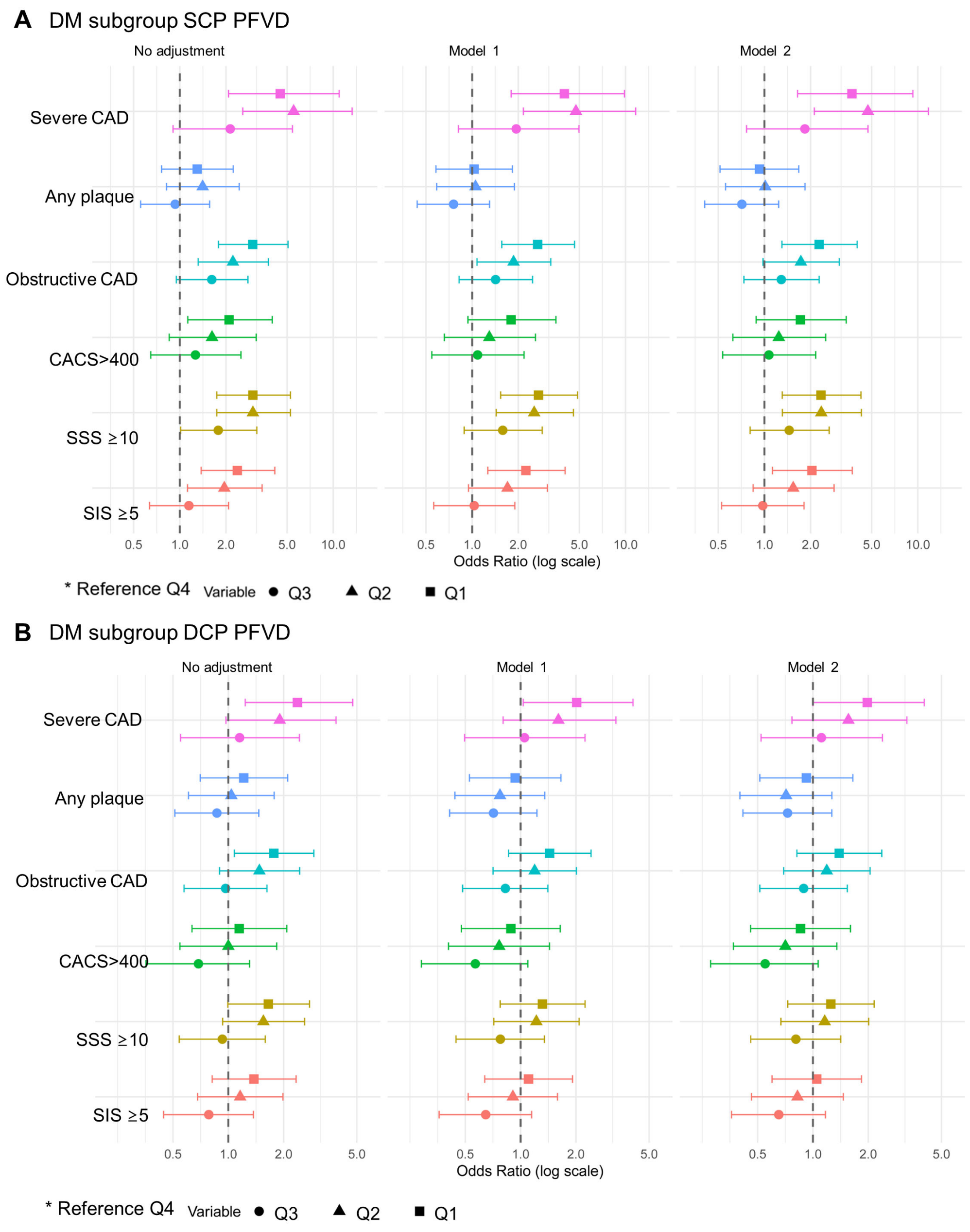

eFigure 15. Association Between Retinal Parafoveal Vascular Density and Subclinical Coronary Atherosclerosis in Participants With Diabetes

(A) Forest plot of logestic regression analyses using quartile of superficial capillary plexus (SCP) parafoveal vascular density (PFVD) and (B) Deep capillary plexus (SCP) PFVD. Reference: Q4 (highest quartile), Model 1: Adjusted for age and sex, Model 2: Adjusted for age, sex, traditional cardiovascular risk factors (hypertension, diabetes, hyperlipidemia, history of smoking, and body mass index categories), and atherosclerotic cardiovascular disease (ASCVD) risk. Abbreviations: CAD, coronary artery disease; CACS, coronary artery calcium score; SIS, segment involvement score; SSS, segment stenosis score

© 2025 Yang JM et al. *JAMA Cardiology.*

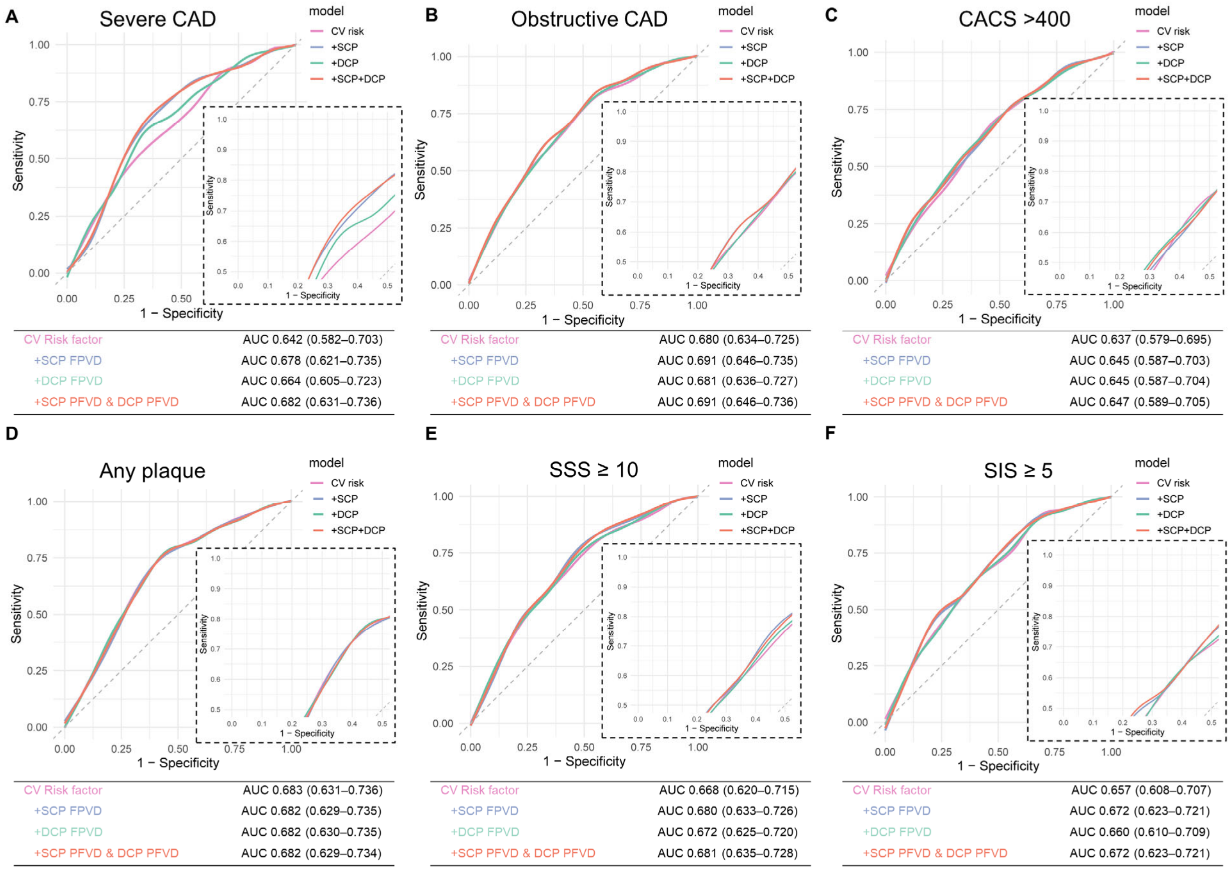

eFigure 16. Incremental Prognostic Value of Parafoveal Vascular Density for Diagnosing Subclinical Coronary Atherosclerosis in Patients With Diabetes

Receiver operating characteristic (ROC) curve for diagnosing subclinical coronary atherosclerosis. The inset panel provides an enlarged view of the ROC curve to aid in visualizing subtle differences between models

© 2025 Yang JM et al. *JAMA Cardiology.*

eTable 1. Relevant Ophthalmic Condition for OCTA Examination

	Overall, n (%)
Purpose of the OCTA examination	1286
GS, n (%)	426 (33.1)
DMR, n (%)	22 (1.7)
NPDR, n (%)	188 (14.6)
PDR, n (%)	67 (5.2)
VH, n (%)	24 (1.9)
RD, n (%)	16 (1.2)
AMD, n (%)	34 (2.6)
dAMD, n (%)	72 (5.6)
wAMD, n (%)	145 (11.3)
MH, n (%)	9 (0.7)
ERM, n (%)	107 (8.3)
CME, n (%)	13 (1.0)
RVO, n (%)	64 (5.0)
CSC, n (%)	30 (2.3)
Uveitis, n (%)	7 (0.5)
Etc, n (%)	134 (10.4)

Abbreviations: AMD, age-related macular degeneration; CME, cystoid macular edema; CSC, central serous chorioretinopathy; dAMD, dry AMD; DMR, diabetic retinopathy; ERM, epiretinal membrane; GS, glaucoma suspect; MH, macular hole; NPDR, non-proliferative diabetic retinopathy; OCTA, optical coherence tomography angiography; PDR, proliferative diabetic retinopathy; RD, retinal detachment; RVO, retinal vein occlusion; VH, vitreous hemorrhage; wAMD, wet AMD

© 2025 Yang JM et al. *JAMA Cardiology*.

eTable 2. Clinical Characteristics of the Participants (Complete Case vs. Missing Case)

Baseline characteristics	Complete case (n = 1286)	Missing case (n = 154)	*P* value	SMD
Age, years (mean (SD))	64.2 (9.9)	64.2 (10.0)	0.86	0.007
Age, years, (n (%))			0.19	0.035
55 <	205 (15.9)	26 (16.9)		
55–70	696 (54.1)	84 (54.5)		
>70	385 (29.9)	44 (28.6)		
Sex, Male, (n (%))	804 (62.5)	102 (66.2)	0.87	0.078
BMI, kg/m^2 (median [IQR])	24.6 [22.6–26.6]	25.0 [23.0–27.2]	0.09	0.070
BMI, kg/m^2 (n(%))				
<25	709 (55.1)	72 (51.8)	0.52	0.096
25–30	503 (39.1)	56 (40.3)		
≥30	74 (5.8)	11 (7.9)		
Smoking, (n (%))			0.99	0.012
Current	135 (10.5)	15 (10.8)		
Never	802 (62.4)	86 (61.9)		
Former	349 (27.1)	38 (27.3)		
Systolic blood pressure, mmHg	130.2 (10.0)	131.0 (9.8)	0.34	0.081
**Comorbidities**				
DM, (n (%))	552 (42.9)	65 (46.8)	0.43	0.077
Hypertension, (n (%))	675 (52.5)	75 (54.0)	0.81	0.029
Hyperlipidemia, (n (%))	930 (72.5)	100 (71.9)	1.00	0.008
Stroke, (n (%))	80 (6.2)	7 (4.5)	0.52	0.074
Kidney disease, (n (%))	94 (7.3)	13 (8.4)	0.73	0.042
**Medications**				
Aspirin, (n (%))	260 (20.2)	31 (20.1)	1.00	0.002
Clopidogrel, (n (%))	337 (26.2)	35 (22.7)	0.40	0.081
Statin, (n (%))	803 (62.4)	97 (63.0)	0.96	0.011
Insulin, (n (%))	108 (8.4)	16 (10.4)	0.50	0.068
Metformin, (n (%))	376 (29.2)	51 (33.1)	0.37	0.084
**Laboratory parameters**				
HbA1c, % (mean (SD))	6.25 (1.18)	6.31 (1.27)	0.91	0.009
<7	992 (77.1)	117 (76.0)	0.09	0.027
≥7	294 (22.9)	37 (24.0)		
Fasting glucose, mg/dL (median [IQR])	111.00 [99.0–135.0]	111.00 [100.0–134.0]	0.81	0.038
TG, mg/dL (mean (SD))	129.3 (75.7)	132.5 (69.7)	0.60	0.045

© 2025 Yang JM et al. *JAMA Cardiology.*

HDL, mg/dL (mean (SD))	50.3 (14.0)	48.6 (12.9)	0.15	0.126
LDL, mg/dL (mean (SD))	102.8 (32.5)	101.2 (31.9)	0.52	0.055
CRP, mg/dL (mean (SD))	0.80 (2.35)	0.86 (2.18)	0.66	0.039
eGFR, mL/min/1.73 m^2 (mean (SD))	85.0 [71.2–95.0]	84.0 [70.2–95.0]	0.84	0.003
≤59	147 (11.4)	17 (11.0)	0.99	0.013
60–89	621 (48.3)	75 (48.7)		
≥90	518 (40.3)	62 (40.3)		
**Ophthalmic and OCTA parameters**				
BCVA, LogMAR (mean (SD))	0.37 (0.44)	0.35 (0.44)	0.45	0.064
FAZ size, mm^2 (mean (SD))	0.35 (0.48)	0.31 (0.14)	0.40	0.092
SCP foveal VD, % (mean (SD))	19.0 (10.2)	18.7 (9.4)	0.81	0.021
SCP parafoveal VD, % (mean (SD))	45.00 (7.32)	45.12 (7.56)	0.86	0.015
DCP fovea VD, % (mean (SD))	32.3 (10.0)	32.5 (9.2)	0.83	0.019
DCP parafoveal VD, % (mean (SD))	49.1 (7.2)	49.2 (6.3)	0.83	0.019
CFT (mean (SD))	264.34 (63.4)	260.3 (57.4)	0.45	0.067
Signal strength (mean (SD))	63.2 (6.9)	62.7 (6.8)	0.39	0.073
**Coronary CTA parameters**				
Coronary artery calcium score (median [IQR])	0 [0–98.7]	1.1 [0–95.4]	0.78	0.086
Coronary artery calcium score, (n (%))			0.72	0.122
0	666 (51.8)	76 (49.4)		
1–10	105 (8.2)	16 (10.4)		
11–100	196 (15.2)	25 (16.2)		
101–400	183 (14.2)	18 (11.7)		
>400	136 (10.6)	19 (12.3)		
Any plaque, (n (%))	804 (62.5)	99 (64.3)	0.75	0.034
Plaque characteristics, (n (%))				
Calcified plaque	685 (53.3)	84 (54.5)	0.91	0.016
Noncalcified plaque	234 (18.2)	27 (17.5)	0.50	0.065
Mixed plaque	320 (24.9)	38 (24.7)	0.22	0.114
Obstructive CAD, (n (%))	300 (23.3)	38 (24.7)	0.78	0.032
Number of obstructive CAD lesions, (n (%))			0.70	0.095
One-vessel	148 (11.5)	17 (11.0)		
Two-vessel	88 (6.8)	10 (6.5)		
Three vessel	63 (4.9)	11 (7.1)		
LM obstruction, (n (%))	23 (1.8)	2 (1.3)	0.91	0.04
Obstructive CAD in the LM or pLAD, (n (%))			0.40	0.123
None	1141 (88.7)	142 (92.2)		
Single	126 (9.8)	10 (6.5)		
Both	19 (1.5)	2 (1.3)		

© 2025 Yang JM et al. *JAMA Cardiology*.

Severe CAD, (n (%))	120 (9.3)	16 (10.4)	0.78	0.036
Segment stenosis score (mean (SD))	5.10 (7.18)	5.30 (7.22)	0.74	0.028
Segment involvement score (mean (SD))	2.53 (2.81)	2.60 (2.88)	0.75	0.027

Abbreviations: BCVA, best-corrected visual acuity; BMI, body mass index; CAD, coronary artery disease; CFT, central foveal thickness; CTA, computed tomography angiography; DCP, deep capillary plexus; DM, diabetes mellitus; FAZ, foveal avascular zone; LM, left main; logMAR, logarithm of the minimum angle of resolution; OCTA, optical coherence tomography angiography; pLAD, proximal left anterior descending; SCP, superficial capillary plexus; SD, standard deviation; SMD, standardized mean difference; VD, vascular density

© 2025 Yang JM et al. *JAMA Cardiology.*

eTable 3. Pearson and Spearman Correlation Coefficients Between OCTA Parameters and Coronary Atherosclerosis Measures

OCTA parameter	CoronaryCTA parameter	Pearson_R	Pearson *P* value	Spearman_rho	Spearman *P* value
**FAZ_size**	Coronary_Calcium_score	0.028	0.31	-0.014	0.62
	Segment stenosis score	0.060	0.03	0.002	0.95
	Segment_involvement_score	0.061	0.03	-0.001	0.98
	No. vessel	0.056	0.04	0.006	0.82
**SCP foveal VD**	Coronary_Calcium_score	0.015	0.58	-0.021	0.46
	Segment stenosis score	-0.045	0.11	-0.075	0.007
	Segment_involvement_score	-0.06	0.03	-0.075	0.007
	No. vessel	-0.018	0.51	-0.026	0.36
**SCP PFVD**	Coronary_Calcium_score	-0.113	<0.001	-0.128	<0.001
	Segment stenosis score	-0.207	<0.001	-0.224	<0.001
	Segment_involvement_score	-0.196	<0.001	-0.212	<0.001
	No. vessel	-0.195	<0.001	-0.223	<0.001
**DCP foveal VD**	Coronary_Calcium_score	-0.028	0.32	-0.041	0.14
	Segment stenosis score	-0.095	<0.001	-0.085	0.002
	Segment_involvement_score	-0.086	0.002	-0.075	0.007
	No. vessel	-0.085	0.002	-0.069	0.01
**DCP PFVD**	Coronary_Calcium_score	-0.118	<0.001	-0.134	<0.001
	Segment stenosis score	-0.187	<0.001	-0.201	<0.001
	Segment_involvement_score	-0.174	<0.001	-0.193	<0.001
	No. vessel	-0.174	<0.001	-0.187	<0.001
**CFT**	Coronary_Calcium_score	0.048	0.09	0.06	0.03
	Segment stenosis score	0.038	0.17	0.037	0.18
	Segment_involvement_score	0.058	0.04	0.045	0.11

© 2025 Yang JM et al. *JAMA Cardiology*.

	No. vessel	0.039	0.17	0.058	0.04

© 2025 Yang JM et al. *JAMA Cardiology*.

eTable 4. Quartile Presentation of Clinical Characteristics

	SCP PFVD						DCP PFVD					
	Q4 (highest)	Q3	Q2	Q1 (lowest)	*P* value	*FDR*	Q4 (highest)	Q3	Q2	Q1 (lowest)	*P* value	*FDR*
n	322	322	321	321			322	322	321	321		
SCP PFVD, % (mean (SD))	53.3 (2.0)	48.1 (1.3)	43.4 (1.65)	35.1 (5.2)			49.8 (5.7)	46.5 (5.7)	44.1 (5.9)	39.6 (7.8)		
DCP PFVD, % (mean (SD))	53.9 (5.0)	50.2 (4.5)	48.0 (6.1)	44.2 (8.7)			56.8 (2.3)	51.6 (1.2)	47.8 (1.1)	40.0 (6.9)		
Age, years (mean (SD))	59.8 (8.9)	62.7 (9.8)	66.8 (9.61)	67.7 (9.2)	<0.001	<0.001	60.3 (9.6)	63.7 (9.2)	65.7(10.4)	67.2 (9.1)	<0.001	<0.001
Age, years, (n (%))					<0.001	<0.001					<0.001	<0.001
<55	93 (28.9)	59 (18.3)	29 (9.0)	24 (7.5)			87 (27.0)	53 (16.5)	44 (13.7)	21 (6.5)		
55–70	190 (59.0)	181 (56.2)	172 (53.6)	153 (47.7)			186 (57.8)	186 (57.8)	158 (49.2)	166 (51.7)		
>70	39 (12.1)	82 (25.5)	120 (37.4)	144 (44.9)			49 (15.2)	83 (25.8)	119 (37.1)	134 (41.7)		
Sex, male, (n (%))	186 (57.8)	217 (67.4)	202 (62.9)	199 (62.0)	0.09	0.17	168 (52.2)	202 (62.7)	219 (68.2)	215 (67.0)	<0.001	<0.001
BMI, kg/m^2 (Median [IQR])	24.9 [22.6–26.6]	24.9 [22.8–26.9]	24.4 [22.7–26.4]	24.4 [22.5–26.3]	0.29	0.36	24.6 [22.6-26.5]	24.8 [23.0–26.6]	25.0 [22.6–26.9]	24.1 [22.5–26.2]	0.12	0.17
BMI, kk/m2 [n(%)]												
<25	169 (52.5)	166 (51.6)	185 (57.6)	189 (58.9)	0.49	0.56	179 (55.6)	172 (53.4)	164 (51.1)	194 (60.4)	0.34	0.48
25–30	133 (41.3)	136 (42.2)	120 (37.4)	114 (35.5)			125 (38.8)	133 (41.3)	135 (42.1)	110 (34.3)		
≥30	20 (6.2)	20 (6.2)	16 (5.0)	18 (5.6)			18 (5.6)	17 (5.3)	22 (6.9)	17 (5.3)		
**Comorbidities**												
Hypertension, (n (%))	130 (40.4)	168 (52.2)	173 (53.9)	204 (63.6)	<0.001	<0.001	144 (44.7)	151 (46.9)	192 (59.8)	188 (58.6)	<0.001	<0.001
Hyperlipidemia, (n (%))	216 (67.1)	232 (72.3)	236 (73.8)	246 (76.9)	0.05	0.13	225 (70.1)	221 (68.6)	242 (75.6)	242 (75.6)	0.09	0.16
Stroke, (n (%))	10 (3.1)	15 (4.7)	26 (8.1)	29 (9.0)	0.005	0.02	11 (3.4)	11 (3.4)	22 (6.9)	36 (11.2)	<0.001	<0.001
Kidney disease, (n (%))	8 (2.5)	21 (6.5)	25 (7.8)	40 (12.5)	<0.001	<0.001	8 2.5)	19 (5.9)	34 (10.6)	33 (10.3)	<0.001	<0.001
**Medications**												
Aspirin, (n (%))	44 (13.7)	62 (19.3)	75 (23.4)	79 (24.6)	0.002	0.008	46 (14.3)	63 (19.6)	66 (20.6)	85 (26.5)	0.002	0.08
Clopidogrel, (n (%))	41 (12.7)	78 (24.2)	109 (34.0)	109 (34.0)	<0.001	<0.001	56 (17.4)	77 (23.9)	90 (28.0)	114 (35.5)	<0.001	<0.001

© 2025 Yang JM et al. *JAMA Cardiology.*

Statin, (n (%))	175 (54.3)	203 (63.0)	204 (63.6)	221 (68.8)	0.002	0.008	183 (56.8)	185 (57.5)	219 (68.2)	216 (67.3)	0.001	<0.001
Insulin, (n (%))	7 (2.2)	25 (7.8)	36 (11.2)	40 (12.5)	<0.001	<0.001	14 (4.3)	20 (6.2)	37 (11.5)	37 (11.5)	0.001	<0.001
Metformin, (n (%))	67 (20.8)	95 (29.5)	104 (32.4)	110 (34.3)	0.001	0.008	74 (23.0)	87 (27.0)	112 (34.9)	103 (32.1)	0.004	<0.001
**Laboratory parameters**												
CRP, mg/dL (mean (SD))	1.07 (3.33)	0.69 (1.93)	0.67 (1.50)	0.83 (2.46)	0.31	0.71	0.99 (3.19)	0.75 (1.65)	0.79 (2.13)	0.71 (2.31)	0.92	0.92
eGFR, mL/min/1.73 m^2 (median [IQR))	91.0 [81.2–99.0]	87.0 [73.2–96.0]	84.0 [69.0–92.0]	80.0 [65.0–91.0]	<0.001	<0.001	91.0 [79.3–98.0]	89.0 [77.0–96.0]	81.0 [66.0–91.0]	81.0 [67.0–91.0]	<0.001	<0.001
eGFR, mL/min/1.73 m^2 n [%))					<0.001	<0.001					<0.001	<0.001
≤59	15 (4.7)	35 (10.9)	42 (13.1)	55 (17.1)			18 (5.6)	31 (9.6)	51 (15.9)	47 (14.6)		
60–89	132 (41.0)	148 (46.0)	165 (51.4)	176 (54.8)			136 (42.2)	137 (42.5)	173 (53.9)	175 (54.5)		
≥90	175 (54.3)	139 (43.2)	114 (35.5)	90 (28.0)			168 (52.2)	154 (47.8)	97 (30.2)	99 (30.8)		

* Multiple comparisons were adjusted using the Benjamini–Hochberg (BH) procedure to control the false discovery rate (FDR)

Abbreviations: DCP, deep capillary plexus; PFVD, parafoveal vascular density; SCP, superficial capillary plexus; SD, standard deviation; HbA1c, glycated hemoglobin; eGFR, estimated glomerular filtration rate

© 2025 Yang JM et al. *JAMA Cardiology.*

eTable 5. Logistic Regression Analysis for Parafoveal Vascular Density and CAD Outcomes

			SCP PFVD				DCP PFVD		
**Outcome**	**Model**	**Variable**	**OR_CI**	***P* value**	***FDR***		**OR_CI**	***P* value**	***FDR***
Any plaque	Model 1	Q3	1.22 (0.87-1.70)	0.25	0.32		1.20 (0.86-1.66)	0.29	0.29
		Q2	1.32 (0.94-1.86)	0.11	0.21		1.52 (1.08-2.14)	0.02	0.04
		Q1	1.58 (1.11-2.24)	0.01	0.03		1.49 (1.05-2.11)	0.03	0.05
	Model 2	Q3	1.08 (0.77-1.53)	0.65	0.65		1.22 (0.87-1.72)	0.26	0.29
		Q2	1.19 (0.83-1.69)	0.35	0.39		1.34 (0.94-1.92)	0.11	0.14
		Q1	1.28 (0.89-1.85)	0.19	0.29		1.36 (0.94-1.95)	0.10	0.14
	No adjustment	Q3	1.56 (1.14-2.13)	0.006	0.02		1.52 (1.11-2.07)	0.009	0.03
		Q2	1.90 (1.38-2.62)	<0.001	<0.001		2.18 (1.58-3.02)	<0.001	<0.001
		Q1	2.36 (1.71-3.28)	<0.001	<0.001		2.35 (1.70-3.26)	<0.001	<0.001
CACS>400	Model 1	Q3	1.05 (0.56-1.99)	0.88	0.88		1.53 (0.85-2.81)	0.16	0.24
		Q2	1.80 (1.02-3.30)	0.05	0.11		1.34 (0.74-2.49)	0.34	0.38
		Q1	2.20 (1.25-3.99)	0.007	0.02		1.92 (1.09-3.49)	0.03	0.08
	Model 2	Q3	0.85 (0.44-1.64)	0.62	0.69		1.45 (0.79-2.71)	0.23	0.3
		Q2	1.39 (0.77-2.61)	0.29	0.43		1.03 (0.55-1.95)	0.93	0.93
		Q1	1.61 (0.89-2.98)	0.12	0.22		1.66 (0.93-3.05)	0.1	0.17
	No adjustment	Q3	1.28 (0.69-2.42)	0.43	0.55		1.88 (1.06-3.44)	0.03	0.08
		Q2	2.40 (1.38-4.31)	0.002	0.01		1.89 (1.06-3.45)	0.03	0.08
		Q1	3.01 (1.76-5.35)	<0.001	<0.001		2.87 (1.68-5.11)	<0.001	0.002
Severe CAD	Model 1	Q3	1.81 (0.79-4.52)	0.18	0.2		1.32 (0.66-2.71)	0.43	0.49
		Q2	5.34 (2.58-12.53)	<0.001	<0.001		2.14 (1.14-4.22)	0.02	0.05
		Q1	4.71 (2.25-11.11)	<0.001	<0.001		2.49 (1.34-4.89)	0.005	0.02
	Model 2	Q3	1.44 (0.62-3.65)	0.41	0.41		1.28 (0.63-2.68)	0.49	0.49
		Q2	4.20 (1.98-10.01)	<0.001	<0.001		1.75 (0.91-3.51)	0.1	0.15
		Q1	3.30 (1.55-7.91)	0.004	0.006		2.10 (1.11-4.17)	0.03	0.05
	No adjustment	Q3	2.19 (0.96-5.43)	0.07	0.09		1.61 (0.82-3.28)	0.17	0.22
		Q2	7.07 (3.48-16.38)	<0.001	<0.001		2.95 (1.60-5.75)	<0.001	0.004
		Q1	6.57 (3.22-15.24)	<0.001	<0.001		3.68 (2.03-7.08)	<0.001	<0.001
Obstructive CAD	Model 1	Q3	2.00 (1.26-3.22)	0.004	0.004		1.32 (0.86-2.04)	0.21	0.23
		Q2	2.75 (1.75-4.40)	<0.001	<0.001		1.83 (1.21-2.80)	0.005	0.01
		Q1	3.74 (2.40-5.95)	<0.001	<0.001		1.96 (1.30-3.00)	0.002	0.005
	Model 2	Q3	1.69 (1.04-2.77)	0.04	0.04		1.32 (0.84-2.08)	0.24	0.24
		Q2	2.27 (1.42-3.71)	<0.001	0.001		1.58 (1.02-2.48)	0.04	0.05
		Q1	2.91 (1.83-4.73)	<0.001	<0.001		1.75 (1.13-2.72)	0.01	0.02
	No adjustment	Q3	2.42 (1.54-3.87)	<0.001	<0.001		1.64 (1.08-2.51)	0.02	0.03
		Q2	3.66 (2.37-5.77)	<0.001	<0.001		2.57 (1.73-3.87)	<0.001	<0.001
		Q1	5.10 (3.34-7.99)	<0.001	<0.001		2.98 (2.01-4.47)	<0.001	<0.001

© 2025 Yang JM et al. *JAMA Cardiology*.

SIS≥5	Model 1	Q3	1.37 (0.82-2.30)	0.23	0.26		1.88 (1.16-3.11)	0.01	0.02
		Q2	2.17 (1.34-3.58)	0.002	0.004		1.72 (1.06-2.86)	0.03	0.04
		Q1	2.62 (1.63-4.31)	<0.001	<0.001		2.11 (1.31-3.48)	0.003	0.006
	Model 2	Q3	1.14 (0.68-1.94)	0.63	0.63		1.91 (1.16-3.21)	0.01	0.02
		Q2	1.77 (1.07-2.98)	0.03	0.04		1.43 (0.86-2.41)	0.18	0.18
		Q1	1.97 (1.20-3.30)	0.008	0.01		1.90 (1.16-3.17)	0.01	0.02
	No adjustment	Q3	1.66 (1.01-2.77)	0.05	0.06		2.31 (1.44-3.78)	<0.001	0.002
		Q2	2.82 (1.78-4.58)	<0.001	<0.001		2.41 (1.51-3.94)	<0.001	0.001
		Q1	3.49 (2.22-5.62)	<0.001	<0.001		3.15 (1.99-5.09)	<0.001	<0.001
SSS≥10	Model 1	Q3	2.49 (1.49-4.28)	<0.001	<0.001		1.90 (1.20-3.08)	0.007	0.009
		Q2	3.99 (2.44-6.79)	<0.001	<0.001		2.08 (1.32-3.36)	0.002	0.003
		Q1	4.01 (2.44-6.84)	<0.001	<0.001		2.45 (1.55-3.93)	<0.001	<0.001
	Model 2	Q3	2.11 (1.24-3.68)	0.007	0.007		1.94 (1.20-3.18)	0.008	0.009
		Q2	3.35 (2.01-5.79)	<0.001	<0.001		1.76 (1.09-2.88)	0.02	0.02
		Q1	3.04 (1.82-5.26)	<0.001	<0.001		2.20 (1.38-3.59)	0.001	0.002
	No adjustment	Q3	3.00 (1.81-5.12)	<0.001	<0.001		2.33 (1.48-3.73)	<0.001	<0.001
		Q2	5.23 (3.24-8.79)	<0.001	<0.001		2.91 (1.87-4.63)	<0.001	<0.001
		Q1	5.48 (3.40-9.19)	<0.001	<0.001		3.66 (2.37-5.77)	<0.001	<0.001

Multiple comparisons were adjusted using the Benjamini–Hochberg (BH) procedure to control the false discovery rate (FDR)

Reference: Q4 (highest quartile), Model 1: Adjusted for age and sex, Model 2: Adjusted for age, sex, and traditional cardiovascular risk factors (hypertension, diabetes, hyperlipidemia, history of smoking, and body mass index categories) and ASCVD risk

© 2025 Yang JM et al. *JAMA Cardiology.*

eTable 6. Odds Ratios per Unit Increase in Parafoveal Vascular Density for Coronary Atherosclerosis Outcomes

Outcome	Model	SCP PFVD				DCP PFVD		
		OR_CI	*P value*	*FDR*		OR_CI	*P value*	*FDR*
Any plaque	Model 2	0.99 (0.97-1.01)	0.25	0.25		0.99 (0.97-1.01)	0.20	0.200
	Model 1	0.98 (0.96-1.00)	0.03	0.04		0.98 (0.96-1.00)	0.05	0.056
	Unadjusted	0.96 (0.94-0.97)	<0.001	0.002		0.96 (0.94-0.97)	<0.001	0.002
CACS>400	Model 2	0.98 (0.95-1.00)	0.06	0.06		0.96 (0.94-0.99)	0.003	0.005
	Model 1	0.97 (0.94-0.99)	0.005	0.007		0.96 (0.94-0.99)	0.002	0.004
	Unadjusted	0.95 (0.93-0.98)	<0.001	0.002		0.95 (0.93-0.97)	<0.001	0.002
Severe CAD	Model 2	0.96 (0.94-0.99)	0.006	0.008		0.97 (0.94-0.99)	0.01	0.013
	Model 1	0.95 (0.93-0.98)	<0.001	0.002		0.96 (0.94-0.99)	0.003	0.005
	Unadjusted	0.94 (0.92-0.96)	<0.001	0.002		0.95 (0.93-0.97)	<0.001	0.002
Obstructive CAD	Model 2	0.96 (0.94-0.98)	<0.001	0.002		0.97 (0.95-0.99)	0.004	0.006
	Model 1	0.96 (0.94-0.97)	<0.001	0.002		0.97 (0.95-0.99)	<0.001	0.002
	Unadjusted	0.94 (0.92-0.96)	<0.001	0.002		0.95 (0.93-0.97)	<0.001	0.002
SIS ≥5	Model 2	0.98 (0.96-1.00)	0.05	0.06		0.98 (0.96-1.00)	0.08	0.085
	Model 1	0.97 (0.95-0.99)	0.002	0.003		0.98 (0.96-1.00)	0.03	0.036
	Unadjusted	0.96 (0.94-0.97)	<0.001	0.002		0.96 (0.94-0.98)	<0.001	0.002
SSS ≥10	Model 2	0.97 (0.95-0.99)	0.002	0.003		0.97 (0.95-0.99)	0.001	0.002
	Model 1	0.96 (0.94-0.98)	<0.001	0.002		0.97 (0.95-0.98)	<0.001	0.002
	Unadjusted	0.94 (0.93-0.96)	<0.001	0.002		0.95 (0.93-0.97)	<0.001	0.002

Multiple comparisons were adjusted using the Benjamini–Hochberg (BH) procedure to control the false discovery rate (FDR)

Reference: Q4 (highest quartile), Model 1: Adjusted for age and sex, Model 2: Adjusted for age, sex, and traditional cardiovascular risk factors (hypertension, diabetes, hyperlipidemia, history of smoking, and body mass index categories) and ASCVD

Abbreviations: ASCVD, atherosclerotic cardiovascular disease; DCP, deep capillary plexus; PFVD, parafoveal vascular density; SCP, superficial capillary plexus;

© 2025 Yang JM et al. *JAMA Cardiology.*

eTable 7. Variance Inflation Factor (VIF) Values of Factors in Logestic Regression Model

SCP PFVD		DCP PFVD	
Variable	VIF	Variable	VIF
**Severe CAD**			
SCP PFVD quartile	1.09	DCP PFVD quartile	1.09
Age	1.14	Age	1.85
Sex	1.40	Sex	3.30
Hypertension	1.13	Hypertension	1.13
Hyperlipidemia	1.03	Hyperlipidemia	1.03
History_Smoking	1.42	History_Smoking	1.40
BMI_cat	1.07	BMI_cat	1.08
DM	1.07	DM	1.20
ASCVD risk	3.79	ASCVD risk	3.79
**Obstructive CAD**			
SCP PFVD quartile	1.08	DCP PFVD quartile	1.08
Age	1.13	Age	1.83
Sex	1.40	Sex	3.07
Hypertension	1.11	Hypertension	1.11
Hyperlipidemia	1.03	Hyperlipidemia	1.03
History_Smoking	1.40	History_Smoking	1.39
BMI_cat	1.08	BMI_cat	1.09
DM	1.07	DM	1.24
ASCVD risk	3.84	ASCVD risk	3.41
**Any plaque**			
SCP PFVD quartile	1.14	DCP PFVD quartile	1.10
Age	1.73	Age	1.70
Sex	3.12	Sex	3.12
Hypertension	1.13	Hypertension	1.13
Hyperlipidemia	1.05	Hyperlipidemia	1.05
History_Smoking	1.56	History_Smoking	1.56
BMI_cat	1.09	BMI_cat	1.09
DM	1.25	DM	1.24
ASCVD risk	2.96	ASCVD risk	2.95
**CACS>400**			
SCP PFVD quartile	1.15	DCP PFVD quartile	1.12
Age	1.97	Age	1.94
Sex	3.00	Sex	2.97
Hypertension	1.15	Hypertension	1.15
Hyperlipidemia	1.06	Hyperlipidemia	1.06
History_Smoking	1.38	History_Smoking	1.37
BMI_cat	1.08	BMI_cat	1.08
DM	1.24	DM	1.23
ASCVD risk	3.63	ASCVD risk	3.63
**SSS ≥10**			

© 2025 Yang JM et al. *JAMA Cardiology.*

SCP PFVD quartile	1.10	DCP PFVD quartile	1.09
Age	1.87	Age	1.85
Sex	3.16	Sex	3.11
Hypertension	1.13	Hypertension	1.12
Hyperlipidemia	1.04	Hyperlipidemia	1.04
History_Smoking	1.41	History_Smoking	1.41
BMI_cat	1.09	BMI_cat	1.08
DM	1.24	DM	1.23
ASCVD risk	3.51	ASCVD risk	3.47
**SIS ≥5**			
SCP PFVD quartile	1.12	DCP PFVD quartile	1.10
Age	1.98	Age	1.96
Sex	2.94	Sex	2.90
Hypertension	1.13	Hypertension	1.12
Hyperlipidemia	1.04	Hyperlipidemia	1.05
History_Smoking	1.36	History_Smoking	1.35
BMI_cat	1.09	BMI_cat	1.09
DM	1.26	DM	1.25
ASCVD risk	3.50	ASCVD risk	3.49

Abbreviations: ASCVD, atherosclerotic cardiovascular disease; BMI, body mass index; CACS, Coronary artery calcium score; DCP, deep capillary plexus parafoveal vascular density; DM, diabetes mellitus; SCP; superficial capillary plexus; SIS, segment involvement score; SSS, segment stenosis score

© 2025 Yang JM et al. *JAMA Cardiology*.

eTable 8. Clinical Characteristics of the Participants Age 30-79

**Baseline characteristics**	**Overall (n = 1207)**
Age, years (mean (SD))	63.2 (8.8)
Age, years, (n (%))	
55 <	202 (16.7)
55–70	695 (57.6)
>70	310 (25.7)
Sex, Male, (n (%))	763 (63.2)
BMI, kg/m^2, n (%)	
median [IQR]	24.6 [22.6–26.6]
<25	661 (54.8)
25–30	474 (39.3)
≥30	72 (6.0)
Smoking, (n (%))	
Current	133 (11.0)
Never	739 (61.2)
Former	335 (27.8)
Systolic blood pressure, mmHg	130.3 (10.0)
PREVENT (%)	7.5 (4.8)
Low (< 5%)	437 (36.2)
Intermediate (5–15%)	688 (57.0)
High (≥ 15%)	82 (6.8)
**Comorbidities**	
DM, (n (%))	524 (43.4)
Hypertension, (n (%))	621 (51.4)
Hyperlipidemia, (n (%))	867 (71.8)
Stroke, (n (%))	68 (5.6)
Kidney disease, (n (%))	85 (7.0)
**Medications**	
Aspirin, (n (%))	238 (19.7)
Clopidogrel, (n (%))	304 (25.2)
Statin, (n (%))	744 (61.6)
Insulin, (n (%))	104 (8.6)
Metformin, (n (%))	360 (29.8)
**Laboratory parameters**	
HbA1c, % (mean (SD))	6.3 (1.2)
Fasting glucose, mg/dL (mean (SD))	123.8 (41.0)
TG, mg/dL (mean (SD))	129.6 (75.0)

© 2025 Yang JM et al. *JAMA Cardiology.*

HDL, mg/dL (mean (SD))	50.3 (14.0)
LDL, mg/dL (mean (SD))	103.5 (32.7)
CRP, mg/dL (mean (SD))	0.79 (2.33)
eGFR, mL/min/1.73 m^2 (median [IQR])	87.0 [73.0–95.0]
**Ophthalmic and OCTA parameters**	
BCVA, LogMAR (mean (SD))	0.35 (0.43)
FAZ size, mm^2 (mean (SD))	0.34 (0.46)
SCP foveal VD, % (mean (SD))	19.1 (10.2)
SCP parafoveal VD, % (mean (SD))	45.2 (7.2)
DCP fovea VD, % (mean (SD))	32.5 (10;0)
DCP parafoveal VD, % (mean (SD))	49.3 (7.0)
CFT (mean (SD))	265.2 (63.3)
**Coronary CTA parameters**	
Coronary artery calcium score (median [IQR])	0 [0–80.6]
Coronary artery calcium score, (n (%))	
0	640 (53.0)
1–10	98 (8.1)
11–100	186 (15.4)
101–400	163 (13.5)
>400	120 (9.9)
Any plaque, (n (%))	740 (61.3)
Plaque characteristics, (n (%))	
Calcified plaque	629 (52.1)
Noncalcified plaque	214 (17.7)
Mixed plaque	288 (23.9)
Obstructive CAD, (n (%))	268 (22.2)
Number of obstructive CAD lesions, (n (%))	
One-vessel	133 (11.0)
Two-vessel	77 (6.4)
Three vessel	57 (4.7)
LM obstruction, (n (%))	20 (1.7)
Obstructive CAD in the LM or pLAD, (n (%))	
None	1082 (89.6)
Single	109 (9.0)
Both	16 (1.3)
Highrisk CAD, (n (%))	105 (8.7)
Segment stenosis score (mean (SD))	4.9 (7.1)
Segment stenosis score ≥ 10	234 (19.4)
Segment involvement score (mean (SD))	2.44 (2.77)

© 2025 Yang JM et al. *JAMA Cardiology*.

Segment involvement score ≥ 5	198 (16.4)

BCVA, best-corrected visual acuity; BMI, body mass index; CAD, coronary artery disease; CFT, central foveal thickness; CTA, computed tomography angiography; DCP, deep capillary plexus; DM, diabetes mellitus; FAZ, foveal avascular zone; LM, left main; logMAR, logarithm of the minimum angle of resolution; OCTA, optical coherence tomography angiography; pLAD, proximal left anterior descending; SCP, superficial capillary plexus; SD, standard deviation; VD, vascular density

© 2025 Yang JM et al. *JAMA Cardiology*.

eTable 9. Comparative AUC Analysis of SCP and DCP Parafoveal Vascular Density (PFVD) in Identifying Subclinical Coronary Atherosclerosis

Outcome	Model (AUC)			*P* value	*P* value	*P* value
	SCP PFVD	DCP PFVD	SCP & DCP PFVD	SCP vs. DCP	SCP vs. SCP & DCP	DCP vs. SCP & DCP.
Severe CAD	0.68 (0.63–0.72)	0.64 (0.60–0.69)	0.68 (0.64–0.73)	0.21\	0.28	**0.035**
Any plaque	0.60 (0.56–0.63)	0.60 (0.56–0.63)	0.60 (0.58–0.64)	0.90	0.12	0.201
Obstructive CAD	0.65 (0.61–0.68)	0.62 (0.59–0.66)	0.66 (0.62–0.69)	0.14	0.13	**0.002**
CACS > 400	0.62 (0.58–0.67)	0.61 (0.56–0.66)	0.623 (0.58–0.67)	0.53	0.93	0.16
SSS ≥ 10	0.65 (0.61–0.68)	0.62 (0.59–0.66)	0.66 (0.62–0.69)	0.22	0.33	**0.002**
SIS ≥ 5	0.62 (0.59–0.66)	0.61 (0.57–0.65)	0.64 (0.60–0.67)	0.49	0.12	0.100

DCP, deep capillary plexus parafoveal vascular density; SCP; superficial capillary plexus; SIS, segment involvement score; SSS, segment stenosis score

© 2025 Yang JM et al. *JAMA Cardiology.*

eTable 10. Diagnostic Performance Metrics of Validation Results With Random Forest

High-risk CAD

Set	AUC	Sens	Spec	PPV	NPV	Acc
Train	0.995 (0.991-0.998)	0.998	0.894	0.989	0.977	0.988
Test	0.757 (0.644-0.866)	0.795	0.949	0.993	0.291	0.809

Obstructive CAD

Set	AUC	Sens	Spec	PPV	NPV	Acc
Train	0.976 (0.967-0.983)	0.907	0.908	0.970	0.749	0.908
Test	0.753 (0.643-0.856)	0.848	0.591	0.874	0.538	0.789

Any plaque

Set	AUC	Sens	Spec	PPV	NPV	Acc
Train	0.961 (0.954-0.974)	0.767	0.632	0.560	0.816	0.683
Test	0.677 (0.555-0.794)	0.757	0.548	0.530	0.789	0.626

CACS400

Set	AUC	Sens	Spec	PPV	NPV	Acc
Train	0.992 (0.988-0.994)	0.912	0.474	0.933	0.403	0.864
Test	0.735 (0.623-0.846)	0.730	0.767	0.966	0.240	0.733

SSS ≥ 10

Set	AUC	Sens	Spec	PPV	NPV	Acc
Train	0.983 (0.976-0.990)	0.861	0.823	0.950	0.601	0.853
Test	0.736 (0.625-0.847)	0.848	0.654	0.908	0.517	0.809

SIS ≥ 5

Set	AUC	Sens	Spec	PPV	NPV	Acc
Train	0.983 (0.975-0.991)	0.754	0.793	0.945	0.405	0.761
Test	0.713 (0.608-0.817)	0.741	0.661	0.913	0.347	0.728

© 2025 Yang JM et al. *JAMA Cardiology*.

eTable 11. Odds Ratios for Low SCP and DCP PFVD in the Full Cohort and in Patients Aged 30–79 Years

Full cohort

Outcome	Model	OR	CI_lower	CI_upper	*P* value	*FDR*
**Low SCP PFVD**						
Obstructive_CAD	No adjustment	2.61	1.99	3.44	<0.001	<0.001
	Model 1	2.09	1.57	2.80	<0.001	<0.001
	Model 2	1.89	1.39	2.57	<0.001	<0.001
Severe_CAD	No adjustment	4.50	2.87	7.36	<0.001	<0.001
	Model 1	3.63	2.27	6.01	<0.001	<0.001
	Model 2	3.13	1.94	5.24	<0.001	<0.001
**Low DCP PFVD**						
Obstructive_CAD	No adjustment	2.22	1.70	2.90	<0.001	<0.001
	Model 1	1.68	1.27	2.23	<0.001	<0.001
	Model 2	1.51	1.13	2.03	0.006	0.009
Severe_CAD	No adjustment	2.64	1.77	4.01	<0.001	<0.001
	Model 1	2.03	1.34	3.12	0.001	0.002
	Model 2	1.74	1.14	2.72	0.012	0.035
**Low SCP & DCP PFVD**						
Obstructive_CAD	No adjustment	2.39	1.83	3.11	<0.001	<0.001
	Model 1	1.89	1.43	2.50	<0.001	<0.001
	Model 2	1.74	1.30	2.33	<0.001	<0.001
Severe_CAD	No adjustment	3.59	2.44	5.33	<0.001	<0.001
	Model 1	2.87	1.93	4.32	<0.001	<0.001
	Model 2	2.54	1.68	3.86	<0.001	<0.001

Model 1: Adjusted for age and sex,

Model 2: Adjusted for age, sex, traditional cardiovascular risk factors (hypertension, diabetes, hyperlipidemia, history of smoking, and body mass index categories), and atherosclerotic cardiovascular disease (ASCVD) risk

© 2025 Yang JM et al. *JAMA Cardiology.*

Patients age (30–79)

Outcome	Model	OR	CI_lower	CI_upper	*P* value	*FDR*
**Low SCP PFVD**						
Obstructive_CAD	No adjustment	2.47	1.87	3.30	<0.001	<0.001
	Model 1	2.03	1.50	2.74	<0.001	<0.001
	Model 2	1.62	1.17	2.24	0.004	0.006
	Model 3	1.67	1.21	2.30	0.002	0.003
Severe_CAD	No adjustment	4.27	2.68	7.09	<0.001	<0.001
	Model 1	3.54	2.18	5.95	<0.001	<0.001
	Model 2	2.78	1.67	4.76	<0.001	<0.001
	Model 3	2.85	1.72	4.90	<0.001	<0.001
**Low DCP PFVD**						
Obstructive_CAD	No adjustment	2.19	1.66	2.90	<0.001	<0.001
	Model 1	1.68	1.26	2.26	<0.001	<0.001
	Model 2	1.37	1.00	1.88	0.052	0.069
	Model 3	1.41	1.03	1.93	0.03	0.05
Highrisk_CAD	No adjustment	2.47	1.63	3.82	<0.001	<0.001
	Model 1	1.94	1.26	3.05	0.003	0.005
	Model 2	1.51	0.96	2.42	0.078	0.19
	Model 3	1.56	0.99	2.49	0.056	0.18
**Low SCP & DCP PFVD**						
Obstructive_CAD	No adjustment	2.29	1.73	3.02	<0.001	<0.001
	Model 1	1.85	1.40	2.47	<0.001	<0.001
	Model 2	1.51	1.10	2.07	0.010	0.010
	Model 3	1.57	1.15	2.15	<0.005	<0.005
Highrisk_CAD	No adjustment	3.26	2.17	4.93	<0.001	<0.001
	Model 1	2.67	1.75	4.09	<0.001	<0.001
	Model 2	2.14	1.37	3.34	<0.001	<0.001
	Model 3	2.21	1.42	3.44	<0.001	<0.001

Model 1: age, sex adjustment

Model 2: CV risk factor (age, sex, hypertension, smoking history, hyperlipidemia, diabetes, BMI category), PREVENT risk

Model 3: CV risk factor (age, sex, hypertension, smoking history, hyperlipidemia, diabetes, BMI category), PREVENT category

Abbreviations: ASCVD, atherosclerotic cardiovascular disease; BMI, body mass index; CAD, coronary artery disease; DCP, deep capillary plexus parafoveal vascular density; SCP; superficial capillary plexus

© 2025 Yang JM et al. *JAMA Cardiology.*

eTable 12. Clinical Characteristics of the Participants Stratified by Diabetes

Baseline characteristics	Non-DM (n = 734)	DM (n = 552)	*P*-value	FDR
Age, years (mean (SD))	63.5 (10.3)	65.2 (9.3)	0.004	0.03
Age, years, (n (%))			0.025	0.08
55 <	129 (17.6)	76 (13.8)		
55–70	405 (55.2)	291 (52.7)		
>70	200 (27.2)	185 (33.5)		
Sex, Male, (n (%))	419 (57.1)	385 (69.7)	<0.001	<0.001
BMI, kg/m^2, n (%)				
median [IQR]	24.2 [22.3–26.2]	25.1 [23.0–27.0]	<0.001	<0.001
<25	441 (60.1)	268 (48.6)	<0.001	<0.001
25–30	265 (36.1)	238 (43.1)		
≥30	28 (3.8)	46 (8.3)		
Smoking, (n (%))			<0.001	<0.001
Current	66 (9.0)	69 (12.5)		
Never	492 (67.0)	310 (56.2)		
Former	176 (24.0)	173 (31.3)		
Systolic blood pressure, mmHg	130.0 (10.0)	130.4 (10.0)	0.42	0.45
ASCVD risk (%)	7.8 (10.1)	18.2 (17.5)	<0.001	<0.001
Low (< 5%)	387 (52.7)	188 (34.1)		
Borderline (5–7.5%)	55 (7.5)	18 (3.3)		
Intermediate (7.5–20%)	212 (28.9)	119 (21.6)		
High (≥ 20%)	80 (10.9)	227 (41.1)		
**Comorbidities**				
Hypertension, (n (%))	301 (41.0)	374 (67.8)	<0.001	<0.001
Hyperlipidemia, (n (%))	479 (65.3)	451 (81.7)	<0.001	<0.001
Stroke, (n (%))	26 (3.5)	54 (9.8)	<0.001	<0.001
Kidney disease, (n (%))	18 (2.5)	76 (13.8)	<0.001	<0.001
**Medications**				
Aspirin, (n (%))	89 (12.1)	171 (31.0)	<0.001	<0.001
Clopidogrel, (n (%))	125 (17.0)	212 (38.4)	<0.001	<0.001
Statin, (n (%))	374 (51.0)	429 (77.7)	<0.001	<0.001
Insulin, (n (%))	0 (0.0)	106 (19.2)	NA	NA
Metformin, (n (%))	0 (0.0)	375 (67.9)	NA	NA
**Laboratory parameters**				
HbA1c, % (mean (SD))	5.6 (0.5)	7.2 (1.3)	<0.001	<0.001
Fasting glucose, mg/dL (median [IQR])	103.0 [96.0–112.0]	135.0 [114.0–167.0]	<0.001	<0.001
Total cholesterol, mg/dL (mean (SD))	172.8 (37.1)	145.0 (37.2)	<0.001	<0.001

© 2025 Yang JM et al. *JAMA Cardiology.*

TG, mg/dL (mean (SD))	126.2 (77.0)	133.3 (73.3)	0.10	0.17
HDL, mg/dL (mean (SD))	53.5 (14.7)	46.1 (12.2)	<0.001	<0.001
LDL, mg/dL (mean (SD))	112.5 (31.9)	90.3 (28.6)	<0.001	<0.001
CRP, mg/dL (mean (SD))	0.71 (2.26)	0.87 (2.29)	0.22	0.26
eGFR, mL/min/1.73 m^2 (mean (SD))	85.2 (15.8)	75.9 (22.0)	<0.001	<0.001
≤59	38 (5.2)	109 (19.7)	<0.001	<0.001
60–89	358 (48.8)	263 (47.6)		
≥90	338 (46.0)	180 (32.6)		
**Ophthalmic and OCTA parameters**				
BCVA, LogMAR (mean (SD))	0.36 (0.44)	0.39 (0.45)	0.15	0.20
FAZ size, mm^2 (mean (SD))	0.33 (0.42)	0.37 (0.54)	0.16	0.20
SCP foveal VD, % (mean (SD))	19.9 (11.0)	17.6 (8.7)	<0.001	<0.001
SCP parafoveal VD, % (mean (SD))	46.0 (7.4)	43.7 (7.0)	<0.001	<0.001
DCP fovea VD, % (mean (SD))	33.8 (10.1)	30.3 (9.6)	<0.001	<0.001
DCP parafoveal VD, % (mean (SD))	49.8 (7.3)	48.2 (6.8)	<0.001	<0.001
CFT (mean (SD))	266.8 (64.1)	261.0 (62.4)	0.10	0.17
**Coronary CTA parameters**				
Coronary artery calcium score (median [IQR])	0 [0–36.1]	9.45 [0–232.7]	<0.001	<0.001
Coronary artery calcium score, (n (%))			<0.001	<0.001
0	429 (58.4)	237 (42.9)		
1–10	61 (8.3)	44 (8.0)		
11–100	117 (15.9)	79 (14.3)		
101–400	91 (12.4)	92 (16.7)		
>400	36 (4.9)	100 (18.1)		
Any plaque, (n (%))	398 (54.2)	406 (73.6)	<0.001	<0.001
Plaque characteristics, (n (%))				
Calcified plaque	342 (46.6)	343 (62.1)	<0.001	<0.001
Noncalcified plaque	107 (14.6)	127 (23.0)	<0.001	<0.001
Mixed plaque	124 (16.9)	196 (35.5)	<0.001	<0.001
Obstructive CAD, (n (%))	107 (14.4)	192 (35.5)	<0.001	<0.001
Number of obstructive CAD lesions, (n (%))			<0.001	<0.001
One-vessel	67 (9.1)	81 (14.7)		
Two-vessel	26 (3.5)	62 (11.2)		
Three vessel	13 (1.8)	50 (9.1)		
LM obstruction, (n (%))	4 (0.5)	19 (3.4)	<0.001	<0.001
Obstructive CAD in the LM or pLAD, (n (%))			<0.001	<0.001
None	688 (93.7)	453 (82.1)		
Single	43 (5.9)	83 (15.0)		

© 2025 Yang JM et al. *JAMA Cardiology*.

Both	3 (0.4)	16 (2.9)		
Severe CAD, (n (%))	31 (4.2)	89 (16.1)	<0.001	<0.001
Segment stenosis score (mean (SD))	3.4 (5.4)	7.4 (8.5)	<0.001	<0.001
Segment stenosis score ≥ 10	87 (11.9)	174 (31.5)	<0.001	<0.001
Segment involvement score (mean (SD))	1.90 (2.48)	3.36 (3.01)	<0.001	<0.001
Segment involvement score ≥ 5	73 (9.9)	147 (26.6)	<0.001	<0.001

Abbreviations: BCVA, best-corrected visual acuity; BMI, body mass index; CAD, coronary artery disease; CFT, central foveal thickness; CTA, computed tomography angiography; DCP, deep capillary plexus; DM, diabetes mellitus; FAZ, foveal avascular zone; LM, left main; logMAR, logarithm of the minimum angle of resolution; OCTA, optical coherence tomography angiography; pLAD, proximal left anterior descending; SCP, superficial capillary plexus; SD, standard deviation; VD, vascular density

© 2025 Yang JM et al. *JAMA Cardiology*.

eTable 13. Coronary Computed Tomography Angiography Parameters by Retinal Parafoveal Vascular Density Quartile in Diabetic Patients

	SCP PFVD						DCP PFVD					
	Q4 (highest)	Q3	Q2	Q1 (lowest)	*P* value	FDR	Q4 (highest)	Q3	Q2	Q1 (lowest)	*P* value	FDR
n	138	138	138	138			138	138	138	138		
SCP PFVD, % (mean (SD))	51.93 (2.33)	46.75 (1.16)	41.85 (1.60)	34.41 (4.66)			47.44 (6.55)	44.87 (5.80)	43.19 (5.13)	39.45 (7.89)		
DCP PFVD, % (mean (SD))	51.93 (5.47)	48.97 (4.61)	47.53 (5.83)	44.23 (8.43)			55.69 (2.40)	50.40 (1.08)	46.97 (1.15)	39.60 (6.45)		
Age, years (mean (SD))	60.8 (9.4)	64.8 (8.6)	68.1 (8.9)	66.9 (8.6)	<0.001	<0.001	62.6 (9.4)	64.1 (8.8)	67.6 (9.1)	66.3 (9.0)	<0.001	<0.001
Age, years, (n (%))					<0.001	<0.001					<0.001	<0.001
<55	39 (28.3)	18 (13.0)	9 (6.5)	10 (7.2)			31 (22.5)	22 (15.9)	13 (9.4)	10 (7.2)		
55–70	77 (55.8)	79 (57.2)	65 (47.1)	70 (50.7)			77 (55.8)	75 (54.3)	66 (47.8)	73 (52.9)		
>70	22 (15.9)	41 (29.7)	64 (46.4)	58 (42.0)			30 (21.7)	41 (29.7)	59 (42.8)	55 (39.9)		
Sex, male, (n (%))	101 (73.2)	101 (73.2)	95 (68.8)	88 (63.8)	0.26	0.48	87 (63.0)	103 (74.6)	99 (71.7)	96 (69.6)	<0.001	<0.001
Smoking, (n (%))					0.49	0.64					0.55	0.70
Current	20 (14.5)	20 (14.5)	13 (9.4)	16 (11.6)			23 (16.7)	16 (11.6)	16 (11.6)	14 (10.1)		
Never	83 (60.1)	72 (52.2)	81 (58.7)	74 (53.6)			80 (58.0)	76 (55.1)	77 (55.8)	77 (55.8)		
Former	35 (25.4)	46 (33.3)	44 (31.9)	48 (34.8)			35 (25.4)	46 (33.3)	45 (32.6)	47 (34.1)		
Systolic blood pressure, mmHg	130.8 (9.8)	131.2 (9.6)	130.7 (9.9)	129.0 (10.7)	0.29	0.49	130.6 (10.1)	129.3 (9.6)	131.8 (10.2)	130.0 (10.2)		
ASCVD (%)	11.9 (12.9)	17.9 (16.5)	22.5 (19.8)	20.3 (18.2)	<0.001	<0.001	11.9 (12.9)	17.9 (16.5)	22.5 (19.8)	20.3 (18.2)	<0.001	<0.001
**Laboratory parameters**												
TG, mg/dL (mean (SD))	131.2 (66.4)	140.3 (82.4)	130.1 (74.2)	131.6 (69.5)	0.64	0.71	132.9 (65.2)	130.0 (72.3)	133.7 (72.2)	136.5 (83.1)	0.91	0.93
HDL, mg/dL (mean (SD))	47.0 (12.5)	44.9 (12.6)	46.4 (11.9)	46.2 (11.9)	0.54	0.66	48.24 (13.43)	47.14 (12.66)	44.75 (11.71)	44.41 (10.68)	0.022	0.14

© 2025 Yang JM et al. *JAMA Cardiology.*

LDL, mg/dL (mean (SD))	91.9 (28.8)	91.2 (31.3)	87.2 (25.0)	91.0 (29.2)	0.54	0.66	90.6 (29.0)	88.0 (22.4)	90.3 (31.4)	92.5 (31.0)	0.63	0.75
**Diabetic retinopathy**					<0.001	<0.001					<0.001	<0.001
No DR	75 (54.3)	67 (48.6)	76 (55.1)	60 (43.5)			77 (55.8)	67 (48.6)	70 (50.7)	64 (46.4)		
NPDR	57 (41.3)	63 (45.7)	44 (31.9)	43 (31.2)			57 (41.3)	62 (44.9)	52 (37.7)	36 (26.1)		
PDR	6 (4.3)	8 (5.8)	18 (13.0)	35 (25.4)			4 (2.9)	9 (6.5)	16 (11.6)	38 (27.5)		
**Coronary CTA parameters**												
Coronary artery calcium score (median [IQR])	6.2 [0-121.0]	3.1 [0–211.3]	48.2 [0-291.2]	7.3 [0-332.7]	0.310\	0.57	203.2 (355.9)	152.0 (308.3)	324.9 (900.6)	330.2 (746.0)	0.042	0.16
Coronary artery calcium score, (n (%))					0.19	0.38					0.79	0.83
0	58 (42.0)	65 (47.1)	52 (37.7)	62 (44.9)			58 (42.0)	62 (44.9)	59 (42.8)	58 (42.0)		
1–10	14 (10.1)	11 (8.0)	8 (5.8)	11 (8.0)			9 (6.5)	15 (10.9)	9 (6.5)	11 (8.0)		
11–100	26 (18.8)	16 (11.6)	21 (15.2)	16 (11.6)			22 (15.9)	19 (13.8)	16 (11.6)	22 (15.9)		
101–400	22 (15.9)	24 (17.4)	30 (21.7)	16 (11.6)			23 (16.7)	23 (16.7)	28 (20.3)	18 (13.0)		
>400	18 (13.0)	22 (15.9)	27 (19.6)	33 (23.9)			26 (18.8)	19 (13.8)	26 (18.8)	29 (21.0)		
Any plaque, (n (%))	98 (71.0)	96 (69.6)	107 (77.5)	105 (76.1)	0.37	0.52	101 (73.2)	97 (70.3)	102 (73.9)	106 (76.8)	0.68	0.76178
Plaque characteristics, (n (%))	88 (63.8)	75 (54.3)	90 (65.2)	90 (65.2)	0.185	0.384						
Calcified plaque	29 (21.0)	30 (21.7)	34 (24.6)	34 (24.6)	0.838	0.838	92 (66.7)	78 (56.5)	81 (58.7)	92 (66.7)	0.175	0.372
Noncalcified plaque	41 (29.7)	43 (31.2)	54 (39.1)	58 (42.0)	0.089	0.289	29 (21.0)	27 (19.6)	37 (26.8)	34 (24.6)	0.463	0.646
Mixed plaque	31 (22.5)	44 (31.9)	54 (39.1)	64 (46.4)	0.367	0.518	48 (34.8)	53 (38.4)	45 (32.6)	50 (36.2)	0.783	0.826
Obstructive CAD, (n (%))	98 (71.0)	96 (69.6)	107 (77.5)	105 (76.1)	<0.001	<0.001	41 (29.7)	40 (29.0)	53 (38.4)	59 (42.8)	0.18	0.37
Number of obstructive CAD lesions, (n (%))					0.056	0.19					0.056	0.19
One-vessel	18 (13.0)	20 (14.5)	18 (13.0)	25 (18.1)			22 (15.9)	18 (13.0)	19 (13.8)	22 (15.9)		
Two-vessel	9 (6.5)	14 (10.1)	21 (15.2)	18 (13.0)			8 (5.8)	17 (12.3)	18 (13.0)	19 (13.8)		
Three vessel	4 (2.9)	10 (7.2)	15 (10.9)	21 (15.2)			11 (8.0)	5 (3.6)	16 (11.6)	18 (13.0)		

© 2025 Yang JM et al. *JAMA Cardiology*.

LM obstruction, (n (%))	4 (2.9)	2 (1.4)	7 (5.1)	6 (4.3)	0.36	0.52	5 (3.6)	2 (1.4)	4 (2.9)	8 (5.8)	0.25	0.45
Obstructive CAD in the LM or pLAD, (n (%))					0.001	0.010					0.065	0.21
None	128 (92.8)	118 (85.5)	99 (71.7)	108 (78.3)			122 (88.4)	117 (84.8)	107 (77.5)	107 (77.5)		
Single	8 (5.8)	18 (13.0)	32 (23.2)	25 (18.1)			12 (8.7)	19 (13.8)	28 (20.3)	24 (7.4)		
Both	2 (1.4)	2 (1.4)	7 (5.1)	5 (3.6)			4 (2.9)	2 (1.4)	3 (2.2)	7 (5.1)		
Severe CAD, (n (%))	8 (5.8)	16 (11.6)	35 (25.4)	30 (21.7)	<0.001	<0.001	15 (10.9)	17 (12.3)	26 (18.8)	31 (22.5)	0.027	0.14
Segment stenosis score (mean (SD))	5.09 (6.37)	6.30 (7.65)	8.74 (9.01)	9.50 (9.85)	<0.001	<0.001	6.85 (8.10)	5.88 (6.89)	7.80 (8.74)	9.09 (9.74)	0.013	0.14
Segment stenosis score ≥ 10 (n (%))	25 (18.1)	39 (28.3)	55 (39.9)	55 (39.9)	<0.001	<0.001	37 (26.8)	35 (25.4)	50 (36.2)	52 (37.7)	0.053	0.19
Segment involvement score (mean (SD))	2.71 (2.59)	2.89 (2.80)	3.83 (3.02)	4.01 (3.37)	<0.001	<0.001	3.36 (2.93)	2.88 (2.65)	3.43 (3.08)	3.77 (3.29)	0.10	0.27
Segment involvement score ≥ 5 (n (%))	26 (18.8)	29 (21.0)	43 (31.2)	49 (35.5)	0.004	0.027	35 (25.4)	29 (21.0)	39 (28.3)	44 (31.9)	0.21	0.43

* Multiple comparisons were adjusted using the Benjamini–Hochberg (BH) procedure to control the false discovery rate (FDR)

Abbreviations: CAD, coronary artery disease; CTA, computed tomography angiography; DCP, deep capillary plexus; LM, left main; OCTA, optical coherence tomography angiography; PFVD, parafoveal vascular density; pLAD, proximal left anterior descending; SCP, superficial capillary plexus; SD, standard deviation;

© 2025 Yang JM et al. *JAMA Cardiology.*

eTable 14. Logistic Regression Analysis for Parafoveal Vascular Density and CAD Outcomes in the DM Subgroup

SCP PFVD

Outcome	Model	Variable	OR_CI	*P* value	FDR
Any plaque	Model 2	Q3	0.73 (0.42-1.27)	0.23	0.76
		Q2	0.71 (0.40-1.26)	0.97	0.97
		Q1	0.92 (0.51-1.65)	0.80	0.97
	Model 1	Q3	0.71 (0.41-1.23)	0.31	0.76
		Q2	0.77 (0.44-1.35)	0.87	0.97
		Q1	0.93 (0.53-1.66)	0.92	0.97
	Unadjusted	Q3	0.87 (0.51-1.46)	0.79	0.97
		Q2	1.04 (0.61-1.78)	0.22	0.76
		Q1	1.21 (0.70-2.10)	0.34	0.76
CACS>400	Model 2	Q3	0.56 (0.28-1.08)	0.85	0.85
		Q2	0.74 (0.39-1.40)	0.54	0.70
		Q1	0.88 (0.47-1.63)	0.12	0.33
	Model 1	Q3	0.57 (0.29-1.09)	0.81	0.85
		Q2	0.76 (0.41-1.44)	0.46	0.70
		Q1	0.88 (0.48-1.64)	0.08	0.33
	Unadjusted	Q3	0.69 (0.36-1.31)	0.49	0.70
		Q2	1.00 (0.55-1.83)	0.14	0.33
		Q1	1.15 (0.63-2.08)	0.02	0.19
Severe CAD	Model 2	Q3	1.12 (0.53-2.40)	0.19	0.19
		Q2	1.59 (0.79-3.29)	<0.001	<0.001
		Q1	2.00 (1.02-4.07)	<0.001	<0.001
	Model 1	Q3	1.05 (0.50-2.24)	0.15	0.16
		Q2	1.60 (0.80-3.30)	<0.001	<0.001
		Q1	2.02 (1.03-4.09)	<0.001	<0.001
	Unadjusted	Q3	1.15 (0.55-2.44)	0.09	0.12
		Q2	1.90 (0.97-3.85)	<0.001	<0.001
		Q1	2.38 (1.24-4.75)	<0.001	<0.001
Obstructive CAD	Model 2	Q3	0.89 (0.52-1.54)	0.38	0.38
		Q2	1.20 (0.70-2.05)	0.06	0.09
		Q1	1.40 (0.82-2.38)	<0.001	0.01
	Model 1	Q3	0.83 (0.48-1.41)	0.21	0.24
		Q2	1.19 (0.71-2.01)	0.03	0.05
		Q1	1.44 (0.86-2.41)	<0.001	0.00

© 2025 Yang JM et al. *JAMA Cardiology.*

	Unadjusted	Q3	0.97 (0.57-1.62)	0.08	0.10
		Q2	1.48 (0.90-2.44)	<0.001	0.01
		Q1	1.77 (1.08-2.92)	<0.001	<0.001
SIS≥5	Model 2	Q3	0.98 (0.53-1.81)	0.94	0.94
		Q2	1.54 (0.84-2.84)	0.16	0.24
		Q1	2.04 (1.13-3.74)	0.02	0.04
	Model 1	Q3	1.03 (0.56-1.90)	0.92	0.94
		Q2	1.70 (0.95-3.10)	0.08	0.14
		Q1	2.24 (1.26-4.04)	0.006	0.03
	Unadjusted	Q3	1.15 (0.63-2.08)	0.65	0.84
		Q2	1.95 (1.12-3.44)	0.02	0.04
		Q1	2.37 (1.38-4.16)	0.002	0.02
SSS≥10	Model 2	Q3	1.45 (0.81-2.64)	0.22	0.22
		Q2	2.35 (1.31-4.28)	0.005	0.007
		Q1	2.34 (1.31-4.26)	0.005	0.007
	Model 1	Q3	1.58 (0.89-2.86)	0.12	0.14
		Q2	2.54 (1.44-4.57)	0.002	0.004
		Q1	2.71 (1.54-4.86)	<0.001	0.002
	Unadjusted	Q3	1.78 (1.01-3.18)	0.05	0.06
		Q2	3.00 (1.74-5.26)	<0.001	<0.001
		Q1	3.00 (1.74-5.26)	<0.001	<0.001

DCP PFVD

Outcome	Model	Variable	OR_CI	*P* value	*FDR*
Any plaque	Model 2	Q3	0.73 (0.42-1.27)	0.26	0.59
		Q2	0.71 (0.40-1.26)	0.25	0.59
		Q1	0.92 (0.51-1.65)	0.78	0.91
	Model 1	Q3	0.71 (0.41-1.23)	0.22	0.59
		Q2	0.77 (0.44-1.35)	0.37	0.74
		Q1	0.93 (0.53-1.66)	0.81	0.91
	Unadjusted	Q3	0.87 (0.51-1.46)	0.59	0.88
		Q2	1.04 (0.61-1.78)	0.89	0.93
		Q1	1.21 (0.70-2.10)	0.49	0.80
CACS>400	Model 2	Q3	0.56 (0.28-1.08)	0.09	0.46
		Q2	0.74 (0.39-1.40)	0.35	0.74
		Q1	0.88 (0.47-1.63)	0.68	0.88
	Model 1	Q3	0.57 (0.29-1.09)	0.09	0.46
		Q2	0.76 (0.41-1.44)	0.40	0.76

© 2025 Yang JM et al. *JAMA Cardiology*.

		Q1	0.88 (0.48-1.64)	0.70	0.88
	Unadjusted	Q3	0.69 (0.36-1.31)	0.26	0.59
		Q2	1.00 (0.55-1.83)	1.00	1.00
		Q1	1.15 (0.63-2.08)	0.65	0.88
Severe CAD	Model 2	Q3	1.12 (0.53-2.40)	0.77	0.91
		Q2	1.59 (0.79-3.29)	0.20	0.59
		Q1	2.00 (1.02-4.07)	0.05	0.45
	Model 1	Q3	1.05 (0.50-2.24)	0.90	0.93
		Q2	1.60 (0.80-3.30)	0.19	0.59
		Q1	2.02 (1.03-4.09)	0.04	0.45
	Unadjusted	Q3	1.15 (0.55-2.44)	0.71	0.88
		Q2	1.90 (0.97-3.85)	0.07	0.46
		Q1	2.38 (1.24-4.75)	0.01	0.36
Obstructive CAD	Model 2	Q3	0.89 (0.52-1.54)	0.68	0.88
		Q2	1.20 (0.70-2.05)	0.50	0.80
		Q1	1.40 (0.82-2.38)	0.21	0.59
	Model 1	Q3	0.83 (0.48-1.41)	0.48	0.80
		Q2	1.19 (0.71-2.01)	0.51	0.80
		Q1	1.44 (0.86-2.41)	0.17	0.59
	Unadjusted	Q3	0.97 (0.57-1.62)	0.89	0.93
		Q2	1.48 (0.90-2.44)	0.13	0.59
		Q1	1.77 (1.08-2.92)	0.02	0.36
SIS≥5	Model 2	Q3	0.65 (0.36-1.17)	0.15	0.69
		Q2	0.82 (0.46-1.46)	0.51	0.82
		Q1	1.05 (0.60-1.84)	0.87	0.87
	Model 1	Q3	0.65 (0.36-1.15)	0.14	0.69
		Q2	0.91 (0.52-1.59)	0.73	0.82
		Q1	1.10 (0.64-1.92)	0.72	0.82
	Unadjusted	Q3	0.78 (0.44-1.37)	0.39	0.82
		Q2	1.16 (0.68-1.98)	0.59	0.82
		Q1	1.38 (0.82-2.34)	0.23	0.69
SSS≥10	Model 2	Q3	0.81 (0.46-1.42)	0.46	0.60
		Q2	1.16 (0.67-2.01)	0.60	0.67
		Q1	1.25 (0.73-2.15)	0.42	0.60
	Model 1	Q3	0.78 (0.44-1.35)	0.37	0.60
		Q2	1.22 (0.71-2.08)	0.47	0.6
		Q1	1.31 (0.77-2.24)	0.31	0.6
	Unadjusted	Q3	0.93 (0.54-1.59)	0.78	0.78

© 2025 Yang JM et al. *JAMA Cardiology*.

		Q2	1.55 (0.93-2.60)	0.09	0.42
		Q1	1.65 (0.99-2.76)	0.05	0.42

Multiple comparisons were adjusted using the Benjamini–Hochberg (BH) procedure to control the false discovery rate (FDR)

Reference: Q4 (highest quartile), Model 1: Adjusted for age and sex, Model 2: Adjusted for age, sex, and traditional cardiovascular risk factors (hypertension, diabetes, hyperlipidemia, history of smoking, and body mass index categories) and ASCVD

Abbreviations: ASCVD, atherosclerotic cardiovascular disease; DCP, deep capillary plexus; PFVD, parafoveal vascular density; SCP, superficial capillary plexus;

© 2025 Yang JM et al. *JAMA Cardiology*.

eTable 15. Incremental Value of Optical Coherence Tomography Angiography Variables Over Clinical Risk Factors for Diagnosing Subclinical Coronary Atherosclerosis in Patients With Diabetes

Model	C-index (95% CI)	P for comparison	Sensitivitiy	Specificity	PPV	NPV
**Severe CAD**						
Cardiovascular risk factors*	0.64 (0.58–0.70)	Reference	0.56	0.65	0.24	0.88
Cardiovascular risk factors*+ SCP PFVD	**0.68 (0.62–0.74)**	**0.02**	0.82	0.52	0.25	0.94
Cardiovascular risk factors*+ DCP PFVD	0.66 (0.60–0.72)	0.12	0.62	0.67	0.27	0.90
Cardiovascular risk factors*+ SCP PFVD + DCP PFVD	**0.68 (0.63–0.74)**	**0.02**	0.80	0.54	0.25	0.93
**Obstructive CAD**						
Cardiovascular risk factors*	0.68 (0.63 –0.72)	Reference	0.79	0.50	0.46	0.82
Cardiovascular risk factors*+ SCP PFVD	0.69 (0.65–0.74)	0.15	0.83	0.46	0.46	0.84
Cardiovascular risk factors*+ DCP PFVD	0.68 (0.64–0.73)	0.66	0.78	0.52	0.47	0.81
Cardiovascular risk factors*+ SCP PFVD + DCP PFVD	0.69 (0.65–0.74)	0.55	0.82	0.48	0.46	0.83
**Any plaque**						
Cardiovascular risk factors*	0.68 (0.63–0.74)	Reference	0.68	0.66	0.85	0.43
Cardiovascular risk factors*+ SCP PFVD	0.68 (0.63–0.73)	0.59	0.72	0.64	0.85	0.45
Cardiovascular risk factors*+ DCP PFVD	0.68 (0.63–0.73)	0.70	0.74	0.61	0.84	0.46
Cardiovascular risk factors*+ SCP PFVD + DCP PFVD	0.68 (0.63–0.73)	0.55	0.72	0.62	0.84	0.44
**CACS>400**						
Cardiovascular risk factors*	0.64 (0.58–0.70)	Reference	0.72	0.53	0.25	0.90
Cardiovascular risk factors*+ SCP PFVD	0.64 (0.59–0.70)	0.35	0.72	0.52	0.25	0.89
Cardiovascular risk factors*+ DCP PFVD	0.64 (0.59–0.70)	0.43	0.73	0.51	0.25	0.90

© 2025 Yang JM et al. *JAMA Cardiology.*

Cardiovascular risk factors* + SCP PFVD + DCP PFVD	0.65 (0.59–0.70)	0.36	0.74	0.50	0.25	0.90
**SSS ≥10**						
Cardiovascular risk factors*	0.67 (0.62–0.72)	Reference	0.79	0.48	0.41	0.83
Cardiovascular risk factors* + SCP PFVD	0.68 (0.63–0.73)	0.12	0.78	0.53	0.43	0.84
Cardiovascular risk factors* + DCP PFVD	0.67 (0.62–0.72)	0.50	0.76	0.52	0.42	0.84
Cardiovascular risk factors* + SCP PFVD + DCP PFVD	0.68 (0.64–0.73)	0.10	0.83	0.47	0.42	0.86
**SIS ≥5**						
Cardiovascular risk factors*	0.66 (0.61–0.71)	Reference	0.878	0.368	0.335	0.892
Cardiovascular risk factors* + SCP PFVD	0.67 (0.62–0.72)	0.077	0.721	0.541	0.363	0.842
Cardiovascular risk factors* + DCP PFVD	0.66 (0.61–0.71)	0.530	0.646	0.602	0.371	0.824
Cardiovascular risk factors* + SCP PFVD + DCP PFVD	0.67 (0.62–0.72)	0.076	0.796	0.472	0.353	0.864

Significant values are in bold.

* Cardiovascular risk factor: traditional risk factor – age, sex, smoking, hypertension, diabetes, hyperlipidemia, body mass index category

Abbreviations: DCP, deep capillary plexus; NPV, negative predictive value; PFVD, parafoveal vascular density; PPV, positive predictive value; SCP, superficial capillary plexus; SIS, segment involvement score; SSS, segment stenosis score

© 2025 Yang JM et al. *JAMA Cardiology.*

eAppendix. STROBE Checklist

STROBE Statement—Checklist of items that should be included in reports of ***cross-sectional studies***

	Item No	Recommendation	Page Number
**Title and abstract**	1	(*a*) Indicate the study's design with a commonly used term in the title or the abstract	3
		(*b*) Provide in the abstract an informative and balanced summary of what was done and what was found	3
**Introduction**			
Background/rationale	2	Explain the scientific background and rationale for the investigation being reported	6
Objectives	3	State specific objectives, including any prespecified hypotheses	6
**Methods**			
Study design	4	Present key elements of study design early in the paper	7
Setting	5	Describe the setting, locations, and relevant dates, including periods of recruitment, exposure, follow-up, and data collection	7
Participants	6	(*a*) Give the eligibility criteria, and the sources and methods of selection of participants	7
Variables	7	Clearly define all outcomes, exposures, predictors, potential confounders, and effect modifiers. Give diagnostic criteria, if applicable	7–10
Data sources/ measurement	8*	For each variable of interest, give sources of data and details of methods of assessment (measurement). Describe comparability of assessment methods if there is more than one group	*7–10, eMethod*
Bias	9	Describe any efforts to address potential sources of bias	10
Study size	10	Explain how the study size was arrived at	eMethod
Quantitative variables	11	Explain how quantitative variables were handled in the analyses. If applicable, describe which groupings were chosen and why	10, eMethod
Statistical methods	12	(*a*) Describe all statistical methods, including those used to control for confounding	9–10
		(*b*) Describe any methods used to examine subgroups and interactions	10
		(*c*) Explain how missing data were addressed	9
		(*d*) If applicable, describe analytical methods taking account of sampling strategy	9–10
		(*e*) Describe any sensitivity analyses	10
**Results**			
Participants	13*	(a) Report numbers of individuals at each stage of study—eg numbers potentially eligible, examined for eligibility, confirmed eligible, included in the study, completing follow-up, and analysed	7,11
		(b) Give reasons for non-participation at each stage	7
		(c) Consider use of a flow diagram	7

© 2025 Yang JM et al. *JAMA Cardiology.*

Descriptive data	14*	(a) Give characteristics of study participants (eg demographic, clinical, social) and information on exposures and potential confounders	11
		(b) Indicate number of participants with missing data for each variable of interest	7
Outcome data	15*	Report numbers of outcome events or summary measures	11–13
Main results	16	(*a*) Give unadjusted estimates and, if applicable, confounder-adjusted estimates and their precision (eg, 95% confidence interval). Make clear which confounders were adjusted for and why they were included	11–13
		(*b*) Report category boundaries when continuous variables were categorized	11–13
		(*c*) If relevant, consider translating estimates of relative risk into absolute risk for a meaningful time period	NA
Other analyses	17	Report other analyses done—eg analyses of subgroups and interactions, and sensitivity analyses	11–13
**Discussion**			
Key results	18	Summarise key results with reference to study objectives	14
Limitations	19	Discuss limitations of the study, taking into account sources of potential bias or imprecision. Discuss both direction and magnitude of any potential bias	16
Interpretation	20	Give a cautious overall interpretation of results considering objectives, limitations, multiplicity of analyses, results from similar studies, and other relevant evidence	14–17
Generalisability	21	Discuss the generalisability (external validity) of the study results	14–17
**Other information**			
Funding	22	Give the source of funding and the role of the funders for the present study and, if applicable, for the original study on which the present article is based	18

*Give information separately for exposed and unexposed groups.

Note: An Explanation and Elaboration article discusses each checklist item and gives methodological background and published examples of transparent reporting. The STROBE checklist is best used in conjunction with this article (freely available on the Web sites of PLoS Medicine at http://www.plosmedicine.org/, Annals of Internal Medicine at http://www.annals.org/, and Epidemiology at http://www.epidem.com/). Information on the STROBE Initiative is available at www.strobe-statement.org.

© 2025 Yang JM et al. *JAMA Cardiology.*
