# Supplementary material for: Subclinical Coronary Atherosclerosis and Retinal Optical Coherence Tomography Angiography
Source: JAMA Cardiol. 2025 Sep 17;10(11):1100–11. doi: 10.1001/jamacardio.2025.3036 (PMC12444650; doi:10.1001/jamacardio.2025.3036)
Supplement: Supplement 2. — Data Sharing Statement [file jamacardiol-e253036-s002.pdf]

Data Sharing Statement

Yang. Subclinical Coronary Atherosclerosis and Retinal Optical Coherence Tomography Angiography. *JAMA Cardiol*. Published September 17, 2025.
doi:10.1001/jamacardio.2025.3036

Data
Data available: Yes
Data types: Deidentified participant data
How to access data: The data will be only shared upon request to the corresponding author (yhyoon@amc.seoul.kr, or seungwlee@amc.seoul.kr).
When available: With publication

Supporting Documents
Document types: None

Additional Information
Who can access the data: researchers whose proposed use of the data has been approved
Types of analyses: for any purpose or for a specified purpose
Mechanisms of data availability: after approval of a proposal
